# Hydrodynamics and water quality of a highly anthropized wetland: the case study of the Massaciuccoli basin (Tuscany, Italy)

**DOI:** 10.1007/s11356-024-33899-2

**Published:** 2024-06-18

**Authors:** Francesca Pasquetti, Stefano Natali, Marco Luppichini, Monica Bini, Nicola Del Seppia, Antonio Delgado-Huertas, Roberto Giannecchini

**Affiliations:** 1 Earth Science Department, University of Pisa, Via S. Maria 53, 56126 Pisa, Italy; 2 CIRSEC - Centre for Climate Change Impact–University of Pisa, Via del Borghetto 80, 56124 Pisa, Italy; 3 Istituto Nazionale Di Geofisica E Vulcanologia (INGV), Via Di Vigna Murata 605, 00143 Rome, Italy; 4 Autorità Di Bacino Distrettuale Dell’Appennino Settentrionale, Via V. Veneto, 1, 55100 Lucca, Italy; 5 Instituto Andaluz de Ciencias de La Tierra, CSIC-UGR, Av. de Las Palmeras, 4, 18100 Armilla, Spain

**Keywords:** Wetlands, Massaciuccoli, Human pressure, Hydrochemistry, Stable isotopes, Multivariate statistics, Tuscany

## Abstract

Owing to increasing anthropogenic impacts, wetlands have suffered a serious environmental decline in recent decades. The sustainable management of these natural resources is fundamental to maintain both the ecosystems and the economic activities. The Lake Massaciuccoli and nearby areas represent one of the largest residual coastal marshy areas in Tuscany (Italy). This wetland is characterized by large-scale and intensive agricultural use and affected by reclamation activities, with consequent problems of erosion, subsidence and lake eutrophication and siltation. In this context, an integrated study combining hydrochemical data (water levels, electrical conductivity, pH, turbidity, major ions, trace metals) and stable isotopes (H, O, S) has been performed in the southernmost part of the basin, to better disentangle processes and interactions between groundwater and surface water and to understand the origin of solutes and their evolution. Our results indicated that both groundwater and surface water have a meteoric origin and that geochemical composition of groundwater is mainly affected by local geological and biological processes. Moreover, surface water is affected by sea water mixing and evapotranspiration/precipitation processes. The impact of agricultural activity and the use of fertilizers on the water quality appears to be limited as regards nitrates, indicating that less intense agricultural practices implemented in recent years have been successful. As regards sulfates, Fe, and Mn, we cannot fully elucidate the mechanisms underlying human influence, but the oscillation of water level and degradation of peat enhanced by reclamation and agriculture activities likely played an important role in controlling the fate of these elements. Overall, these results underline the importance of integrated approaches to disentangle geochemical processes and will be useful in supporting policy implementation and environmental protection in this valuable area of Tuscany. Findings from this work suggest the need for policy-making authorities to take actions as soon as possible to mitigate risks. Closer co-operation is essential between authorities and farmers to reduce inputs of fertilizers and chemicals into the lake and the surrounding area. Also, additional policy measures should be enforced to reduce the mechanical soil tillage and limit erosion and runoff, such as the NBSs implemented within the Phusicos Project.

**Supplementary Information:**

The online version contains supplementary material available at 10.1007/s11356-024-33899-2.

## Introduction

Coastal lagoons, lacustrine areas, and wetlands are valuable ecosystems characterized by high biodiversity and productivity. They usually provide essential ecological services, such as key habitats for migratory species and nurseries for aquatic and terrestrial life, also producing food and energy for human use (Newton et al. 2018; Nayak and Bhushan 2022). Nevertheless, in the last decades these ecosystems have suffered a serious decline and environmental degradation worldwide due to increasing anthropogenic impacts, such as urbanization, excessive land use and drainage, and pollution due to agricultural activities (Jones and Hughes 1993; Datta et al. 2022).

The Lake Massaciuccoli (7 km^2^ wide and about 2 m deep) and nearby areas represent one of the largest and most important residual coastal marshy areas in Tuscany (Italy) (Viciani et al. 2017). Considering the ecological value of this wetland, it was designated as Ramsar area (code ITE12W0400; DPR n. 448, 13/03/1976) and included in the Tuscan regional park “Migliarino-San Rossore-Massaciuccoli”. Moreover, the Lake Massaciuccoli area is part of the Natura 2000 network (code IT5120021), has been included in the list of the Sites of Community Importance (code IT5120017; Dir. 92/43/EEC) and designated as an Important Bird Area (IBA 077) according to BirdLife International.

From the other side, in the Massaciuccoli basin important economic activities are also developed. Since 1930, a large part of this basin has been drained for agricultural purposes (Silvestri et al. 2012). Agriculture is traditionally oriented toward cereals and industrial and horticultural crops, with a presence of woody crops, especially olive and peach groves (Silvestri et al. 2012). To ensure a water table depth suitable for cultivation in this palustrine area, a complex network of artificial drains and pumping stations has been used to drain precipitation and the superficial aquifer into the Lake Massaciuccoli. As final water receptor, this lake has become a sensitive and vulnerable area to nutrients, such as nitrates and phosphates, and silting phenomena, which have favored eutrophication conditions and ecosystems degradation (Pensabene et al. 1997; Giannini et al. 2017, 2018; Silvestri et al. 2017). Historically, eutrophication has been one of the main issues of the lake triggered by the abundance of nutrients in the aquatic environment due to human activities in the surroundings areas. Sewage wastewater discharged from residential buildings and productive processes, along with peat mineralization and agricultural land use represent the main factors inducing eutrophication (Ciurli et al. 2009; Lastrucci et al. 2017). This is why from 2003, it has been designated as a nitrate vulnerable zone according to the European directive 91/676/CEE. Moreover, due to land reclamation and oxidation of peat organic soils, this area is experiencing significant land subsidence (Di Grazia et al. 2009). The compaction of soil due to water removal and the reduction of peat mass due to oxidation of peat organic soil led to a lowering of the areas around the lake that is now perched (Di Grazia et al. 2009). This subsidence, among others, has impacts on the maintenance and operational cost of the existing hydraulic infrastructures (e.g., maintenance of the embankments and strengthening of pumping) and on the stability of the buildings of the area (Baldaccini 2018).

In the last decades, several studies and actions were developed to restore and preserve this area. The latest ones date back to 2008–2009 when studies on water quality, hydraulic modeling, and agricultural practices were carried out (Baneschi et al. 2013). In 2020, this area was selected as a case study for the Phusicos Project, funded by the EU Horizon 2020 program and aimed at the identification, application, and monitoring of Nature-Based Solutions (NBSs) able to restore pristine conditions according to the EU Water Framework and Floods Directives (Solheim et al. 2021) and the Sendai Framework for Disaster Risk Reduction 2015–2030 (UNDRR). In the Massaciuccoli basin, the NBSs have been tested to limit soil and nutrient loss from cultivated fields and to treat superficial waters from the ditches network before flowing into the lake (Barsanti et al. 2021; Pignalosa et al. 2022).

In this context, an updated assessment of the general hydrological and chemical-physical conditions of surface water within the drained areas was fundamental to support policy implementation and environmental protection and developing information for scientific reporting. Moreover, to best of our knowledge, no investigations were carried out on groundwater in the Massaciuccoli Lake basin. Therefore, the purpose of this paper is to provide the results of environmental monitoring and surveys carried out from October 2020 to October 2021 on surface water and groundwater of the drained areas located in the south-eastern part of the Lake Massaciuccoli area, which included the characterization of field parameters (water levels, electrical conductivity, pH, turbidity), major ions, trace metals, and stable isotopes (H, O, S). In this study, an integrated approach combining hydrochemical data interpreted by means of multivariate statistics and stable isotope has been performed to better disentangle processes and interactions between groundwater and surface water and to understand the origin of solutes and their evolution.

## Study area

**Fig. 1:**
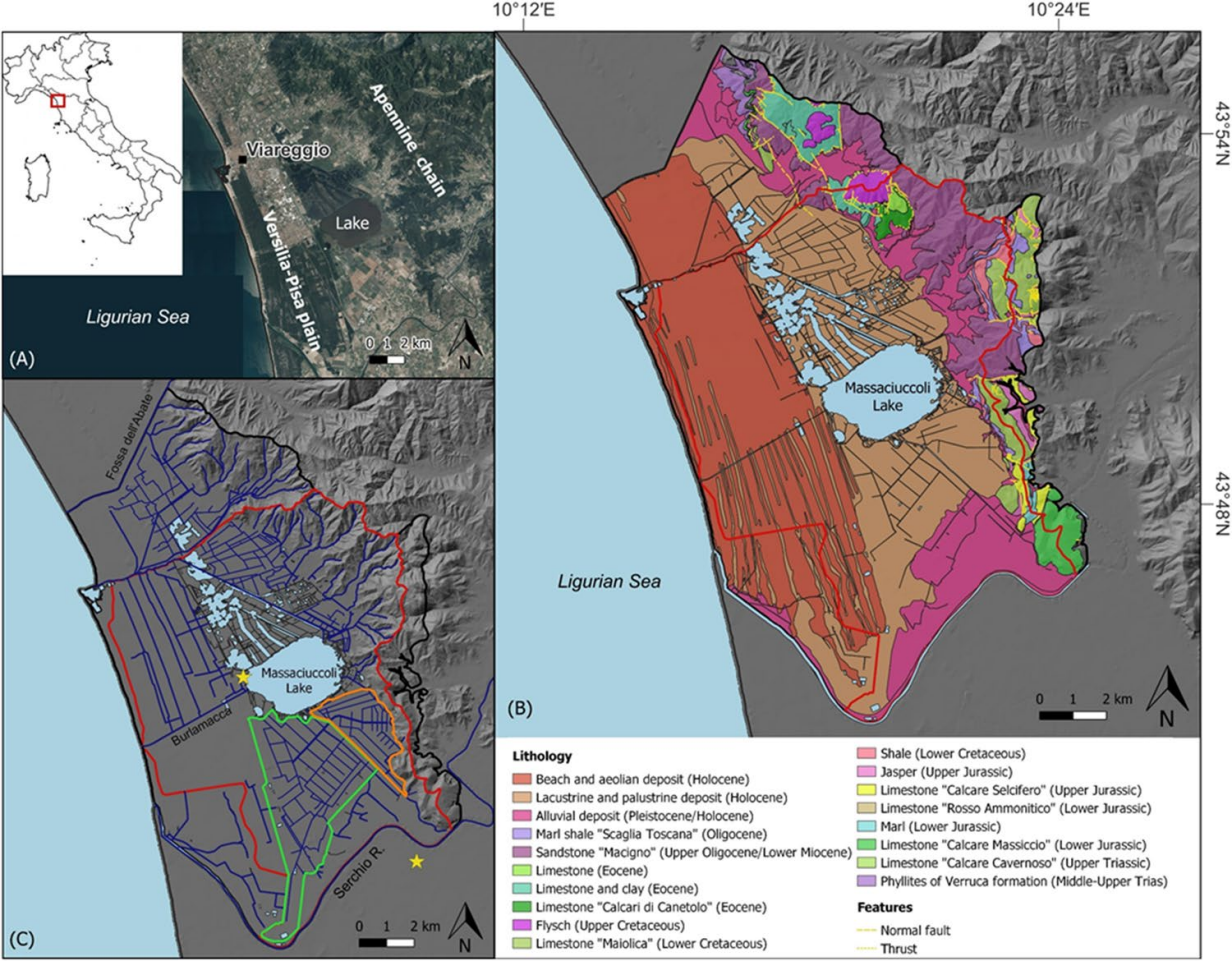
**A** Study area and geographical location of the Lake Massaciuccoli; **B** geological map of the Massaciuccoli basin (Tuscan Geological Database at 1:10000 scale available as shapefile on http://www502.regione.toscana.it/geoscopio). The base of the map is constituted by DTM hillshade effect; **C** hydrological network of the Massaciuccoli basin. The map shows the hydrogeological basin (black line), the hydrological basin (red line), and the two drained areas: the Vecchiano (green line) and Massaciuccoli (orange line) sub-catchments (data available on the website of the Northern Apennines River Basin District Authority https://www.appenninosettentrionale.it/itc). The stars indicate the location of the two meteorological stations of the Regional Hydrologic Service: TOS02004081, near the lake, and TOS11000001, about 6 km away

The Lake Massaciuccoli and nearby marshy areas are located between the Versilia-Pisa plain (northwestern Tuscany, Italy), a coastal plain in front of the Ligurian Sea and delimited eastward by the Apennine chain (Fig. 1A). From a geological point of view, the Versilia-Pisa plain is part of a tectonic basin (called Viareggio Basin) originated from the extensional tectonic phase (Upper Miocene) that followed the Apennine chain formation (Carmignani and Kligfield 1990). This basin is filled with marine, transitional, and continental deposits constituting a stratigraphic sequence over 2000 m thick (Mazzanti and Pasquinucci 1983; Pascucci 2005). This stratigraphic sequence is mainly composed of alternate sands and clays resting unconformably on the Oligocene-early Miocene Macigno sandstone (Tuscan Nappe) (Pascucci 2005). In the area surrounding the lake, surface sediments are mainly constituted by lacustrine and swamp (peat) deposits (Fig. 1B). Alluvial fan sediments outcrop in the foothills, while marine and eolian deposits can be found in the coastal area (Federici 1993; Bini et al. 2013; Luppichini et al. 2022). In the hilly area, outcropping formations are mainly composed of sandstones-siltstones (Macigno Fm.) and argillites (Scaglia Toscana Fm.), whereas in the southern part limestones such as “Calcare Massiccio,” “Calcari Selciferi,” “Calcare Cavernoso,” and “Maiolica” formations prevail (Fig. 1B).

**Fig. 2:**
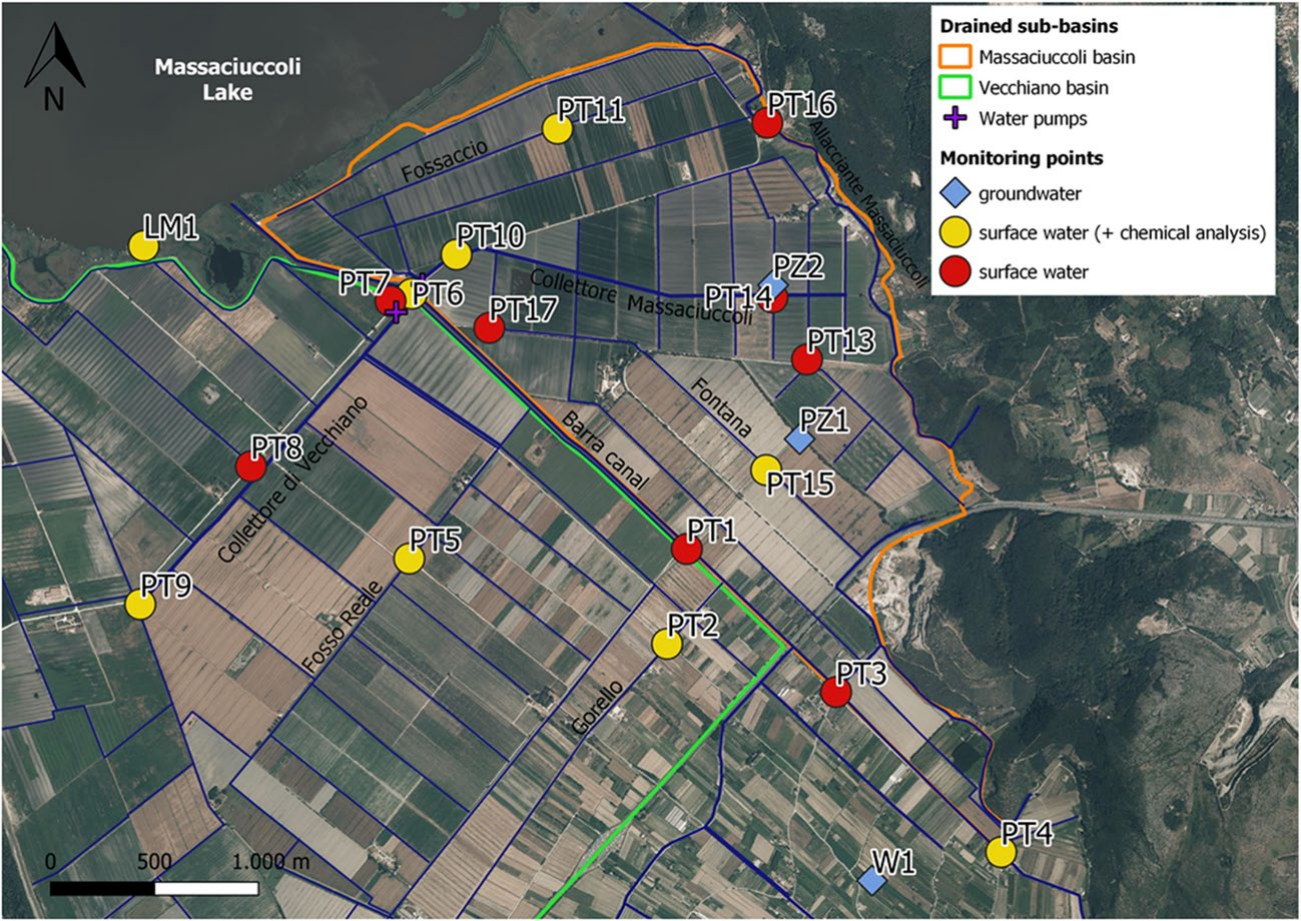
Location of monitoring points for surface water and groundwater. The drainage network is highlighted in blue. The base of the map is an ESRI World imagery

The drainage basin of the Lake Massaciuccoli is about 114 km^2^ and includes the naturally drained hilly area and the coastal plain between Fossa dell’Abate stream to the north and the Serchio River to the south (Fig. 1C). Much of the area surrounding the lake has been reclaimed since 1930 for agricultural purposes by means of a complex network of artificial ditches and pumping stations forcing water from the drained areas into the lake. The drained area in the south-eastern part of the lake is constituted by two sub-catchments, namely: the Vecchiano and Massaciuccoli basins (Fig. 1C). Each basin has a network of ditches collected in two main canals (“Collettore di Vecchiano” and “Collettore Massaciuccoli”, respectively) at the end of which the pumping stations are located (Fig. 2). The Barra canal, the most important hydraulic collector of the area, receives water from the two pumping stations and is the main tributary of the lake (Fig. 2). The Lake Massaciuccoli is also connected to the sea through the Burlamacca canal (Fig. 1C), which is the main outlet, but occasionally, a flux of marine water occurs toward the lake contributing to salinization of water (Baneschi 2007).

The artificial network drains the superficial unconfined aquifer and the runoff associated to excess of rainfall, maintaining a water table depth suitable for cultivation. However, in summer, the water flow direction can be inverted from the lake to cropland to supply irrigation. The superficial aquifer is formed of sandy deposits, which locally reach a thickness of 30–40 m (Rossetto et al. 2010). This aquifer is fed by precipitation infiltrating in the coastal plain and the hilly area and occasionally by infiltration from the Serchio River (Baneschi 2007; Rossetto et al. 2010). Moreover, the peatland subsidence (2–3 m in 70 years; Baneschi et al. 2013) started after land reclamation leaved the lake perched above the drained area determining seepage also from the lake to the superficial aquifer (Rossetto et al. 2010). Marine intrusion from the coast is instead hindered by a piezometric high in the sandy coastal shallow aquifer that acts as a barrier to seawater (Baneschi 2007; Doveri et al. 2009; Rossetto et al. 2010).

According to Köppen’s classification system (Köppen 1931), the climate is explained as Csa in the plain and Csb in the mountain area. Two peaks of precipitation characterize the average rainfall regime: the main one in autumn and the secondary in late winter/spring (Rapetti and Vittorini 1994; Bartolini et al. 2018), whereas summer is the driest season. Mean annual precipitation (MAP) is about 940 mm, as calculated by the instrumental time series from 1996 to 2023 registered at the lake meteorological station (TOS02004081, Torre del Lago, 1 m a.s.l.) of the Regional Hydrologic Service (SIR, https://www.sir.toscana.it). Mean annual temperature (MAT) is about 15.2 °C, as calculated in the period 1990–2023 at the closest meteorological station (TOS11000001, Metato, 3 m a.s.l.), about 6 km away from the lake (Fig. 1C).

## Material and methods

### Monitoring points and collected data

To evaluate the hydrological and chemical-physical conditions of surface water, we performed a monthly monitoring of the main ditches of each sub-basin from October 2020 to October 2021. The monitoring points (denominated “PT”) were selected based on accessibility and the presence of geo-referenceable structures (e.g., bridges): PT2, PT5, PT8, and PT9 were selected in the Vecchiano sub-basin; PT10, PT11, PT13, PT14, PT15, and PT17 were selected in the Massaciuccoli sub-basin; PT1, PT3, PT4, and PT6 were selected on the Barra canal; PT7 is located near the water pumps on the lake side, and PT16 was selected on a stream (Allacciante Massaciuccoli) connected to the lake and located on the border of the Massaciuccoli sub-basin (Fig. 2). For each point water level, flow direction, electrical conductivity (EC), temperature (*T*), pH, and turbidity were monthly measured by portable instruments. In addition, three sampling surveys were carried out on selected points (PT2, PT4, PT5, PT6, PT9, PT10, PT11, PT15) in December 2020, June 2021, and October 2021 to chemically characterize water and then analyze the main cations, anions, and trace elements. During the June 2021 survey, samples for isotope analysis (H and O stable isotopes of water and S and O stable isotopes of dissolved sulfates) were also collected for the monitoring points located on the main ditch of each sub-basin (PT9 and PT10) and the Barra canal (PT4). During the same survey, we also collected samples from the Lake Massaciuccoli (LM1; Fig. 2).

To evaluate the hydrological and chemical-physical conditions of groundwater and the relationship between surface water and the unconfined aquifer, we also performed groundwater monitoring and sampling. The water level data were collected using portable instruments at approximately fortnightly intervals on two piezometers (PZ1 and PZ2; Fig. 2) installed on purpose. PZ1 was realized in September 2020 and consists of a PVC pipe with a diameter of 4.5 cm and a depth of 4.85 m from ground level. The pipe is sealed at the bottom and for the last 0.5 m, while the rest of the pipe (4.35 m) is the screen (scheme in Fig. S1A of the Supplementary material). PZ2 was realized in September 2021 and consists of a PVC pipe with a diameter of 4.5 cm and a depth of 8 m from ground level. The pipe is sealed at the bottom and for the last 0.5 m, whereas the screen is 7.5 m long (scheme in Fig. S1B of Supplementary material). Along the screened portion of the pipe, both piezometers intercept mostly layers of peat, fine sand, and silt of different thickness (Figs. S1A and S1B).

For PZ1 the monitoring period was from September 2020 to September 2022 while for PZ2 was from September 2021 to September 2022. Groundwater from PZ1 was collected during the three sampling surveys for water chemical characterization, while PZ2 was sampled only during the last survey in October 2021. During the three sampling surveys, a well (W1) located outside the drained basins (Fig. 2) and attested in the unconfined aquifer (at a depth of about 7 m) was also sampled for groundwater characterization. Moreover, PZ1 and W1 were included in the sampling for stable isotope analysis.

### Field measurements and sample collection

For each monitoring point, the altitude (m a.s.l.) of the reference structure was measured using a differential GPS (Leica) with real-time kinematic (RTK) positioning (precision ± 10 cm). The water level was then measured using a freatimeter OTT KL 010 (Corr-Tek Idrometria s.r.l.) from the reference point to the water table. For surface water, EC (µS/cm at 25 °C), T (°C), pH, and turbidity (NTU) were measured on water collected by a beaker at the center of the ditch at a depth of about 10–20 cm from the water surface. EC, T, and pH were measured by means of portable conductivity and pH meters (XS instruments) while turbidity was measured through a portable nephelometer AL255T-IR (AQUALYTIC®). The accuracy was ± 1% for EC, ± 0.1 °C for T, 0.02 for pH, and ± 1% for turbidity.

For chemical analysis, two 50 mL aliquots of water were collected in high-density polyethylene (HDPE) bottles. For surface water, samples were collected as previously described, while groundwater samples were collected after a purge of a few minutes. The aliquot for major cations and trace element analyses was filtered through 0.45 µm nylon filters and acidified using ultrapure HNO_3_ to pH lower than 2. The aliquot for major anions analysis was only filtered. Alkalinity (attributable to HCO_3_ˉ, given the pH values) was determined in situ by acidimetric titration with 0.1 N HCl using methyl-orange as an indicator.

For isotope analysis two aliquots of water (50 mL for H and O isotopes of water and 500 mL for S and O isotopes of dissolved sulfates) were filtered through 0.45 µm nylon filters and collected in double sealing HDPE bottles.

All samples were kept at a temperature of about + 4 °C before the analysis.

### Laboratory analysis

Major and trace elements were determined at the Geochemistry laboratory of the Earth Science Department of the University of Pisa. Major ions were determined by ion chromatography using a Thermo ICS 900. For the cations, a Dionex IonPac CS12A-5 μm analytical column was used with the CMMS 300 suppressor. A Dionex IonPac AS23 analytical column along with the ASRS 500 suppressor was used for the anions. Analytical precision, calculated on five replicate injections and expressed as relative standard deviation (RSD), was < 5%. To ensure the accuracy of the analysis the ion balance between major anions and cations was used (Appelo and Postma 2005). For each sample the accuracy was < 10%, except for PT4 in October 2021 (due to anion deficiency) which was then removed from the dataset.

Trace elements (Li, Be, V, Cr, Mn, Fe, Co, Ni, Cu, Zn, As, Sr, Mo, Ag, Cd, Sn, Sb, Ba, Tl, Pb, Th, and U) were determined by inductively coupled plasma mass spectrometry (ICP-MS) using a Perkin Elmer NexION 300X. ^103^Rh, ^187^Re, and ^209^Bi were used as internal standards to correct for signal fluctuations and matrix effects. Trace element concentration determined in the certified reference solution IV-STOCK-1643 (Inorganic Ventures), in five-repeated analyses, were used to evaluate analytical uncertainties. The accuracy and precision were generally < 10%. The detection limits (DL) for each element were evaluated as the mean value of the blank solution concentration (ten replicates) plus three times the standard deviation.

The ^18^O/^16^O and ^2^H/H isotopic ratios of water samples were determined at the Laboratory of Fluid Geochemistry of the University of Florence by cavity ring-down spectroscopy (CRDS) using a Picarro L2130-i analyzer. Picarro’s ChemCorrect post-processing software was used to analyze the spectral features of samples and to determine whether the analysis was compromised by organic molecules. Four internal standards spanning isotope scales of interest were run through the analysis, which were previously calibrated to the VSMOW-SLAP scale. Data were expressed as δ‰ compared to the international reference standard V-SMOW. The analytical precision was within ± 0.1 ‰ for δ^18^O and ± 1 ‰ for δ^2^H. Deuterium excess (*d*-excess) was calculated by Dansgaard’s equation (Dansgaard 1964) using the relationship *d*-excess = δ^2^H – 8xδ^18^O. Error propagation for *d*-excess was ± 1.8 ‰.

The ^34^S/^32^S and ^18^O/^16^O isotopic ratios in dissolved sulfates (SO_4_) were measured at the Stable Isotope Biogeochemistry Laboratory of the Andalusian Earth Sciences Institute (IACT), which belongs to the Spanish Research Council (CSIC) and the University of Granada (UGR). Water samples were acidified to pH < 2 by adding HCl; then the dissolved sulfate was precipitated as barium sulfate (BaSO_4_) by adding a barium chloride (BaCl_2_) solution. The S isotopes were analyzed by combusting the samples with V_2_O_5_ and O_2_ at 1030 °C in a Carlo Erba NC1500 Elemental Analyzer online with a Delta Plus XP mass spectrometer (EA-IRMS). Commercial SO_2_ was used as the internal working standard for analysis of δ^34^S. Three internal standards and IAEA international reference materials were used for calibration. The δ^18^O-SO_4_ was measured with a Thermo Finnigan TC-EA high-temperature pyrolysis system coupled to a Delta Plus XL mass spectrometer. The δ^34^S-SO_4_ and δ^18^O-SO_4_ were expressed relative to the standard reference material V-CDT (Vienna—Canyon Diablo Troilite) and V-SMOW, respectively. The analytical error (2σ) was ± 0.3 ‰ for δ^34^S and ± 0.4 ‰ for δ^18^O.

### Compositional data analysis

Since compositional data are characterized by constant-sum closures (i.e., single components represent proportion of a whole), to investigate reliable and unbiased multivariate relationships between variables constituting the water geochemical composition, compositional data analysis was performed (Aitchison 1986; Buccianti and Grunsky 2014). In particular, the compositional *clr-*biplot (*centered log-ratio* biplot), the geometric center, and the variation array of the dataset were realized and calculated using the CoDapack software v2.03.01 (Comas-Cufi and Thió-Henestrosa 2011). In a *clr-*biplot, the 2-dimensional space is generated by the two first vectors of an *olr*-basis (*orthonormal log-ratio*) that are respectively represented by the horizontal and vertical axes (ilr.1 and ilr.2) (Gabriel 1971; Gower and Hand 1996). A *clr-*biplot can show simultaneously the *clr*-variables and the samples. The *clr-*variables for a composition ***x*** = (*x*_1_, *x*_2_, …, *x*_D_) with D components or variables are defined as:

$$clr \left(\mathbf{x}\right)=\left(\text{log}\frac{{x}_{1}}{{g}_{m}(\mathbf{x})} ,\text{log}\frac{{x}_{2}}{{g}_{m}(\mathbf{x})},\dots ,\text{log}\frac{{x}_{D}}{{g}_{m}(\mathbf{x})}\right)$$

where ${g}_{m}\left(\mathbf{x}\right)$ stands for the geometric mean of the row (Aitchison 1986). In a *clr-*biplot, the *clr-*variables are represented by rays, the end of which is called vertex. The length of a ray provides information on the variability of that variable among samples (i.e., the longest the ray, the higher the variability). The segment from a vertex to another vertex is called link and its length indicates the variance of the log ratio among the variables considered (as also reported in the variation array). A low variance of the log ratio (i.e., a short link) suggests that *clr-*variables are proportional, and thus they may carry the same information (e.g., they can be related to the same environmental process). Samples are represented by points. The distribution of samples in the positive or negative part of the ilr axes indicates which *clr-*variables prevail in those samples. Close samples indicate similar compositions. Moreover, the closer the sample to the center of biplot, the more similar the sample is to the geometric center of the dataset (i.e., the composition more representative of the whole population). On the contrary, samples far away from the center can be possible outliers.

To perform this type of analysis samples or variables containing censored data (e.g., values below detection limit) should be deleted or replaced by data with different imputation methods. In general, if the percentage of censored data is < 10% of the entire dataset, a “simple substitution” is appropriate (Palarea-Albaladejo and Martín-Fernández 2015). One reasonable option is to use a value equal to 65% of the detection limit or of the minimum value for that variable (Martín-Fernández et al. 2003). If the percentage of censored data is > 10% multivariate methods should be applied (Palarea-Albaladejo and Martín-Fernández 2015). In this study, variables containing a percentage of censored data higher than 10% were disregarded; otherwise, a simple substitution was performed using a value equal to 65% of the detection limit for each variable.

## Results

### Hydrodynamic conditions of surface water and groundwater

**Fig. 3:**
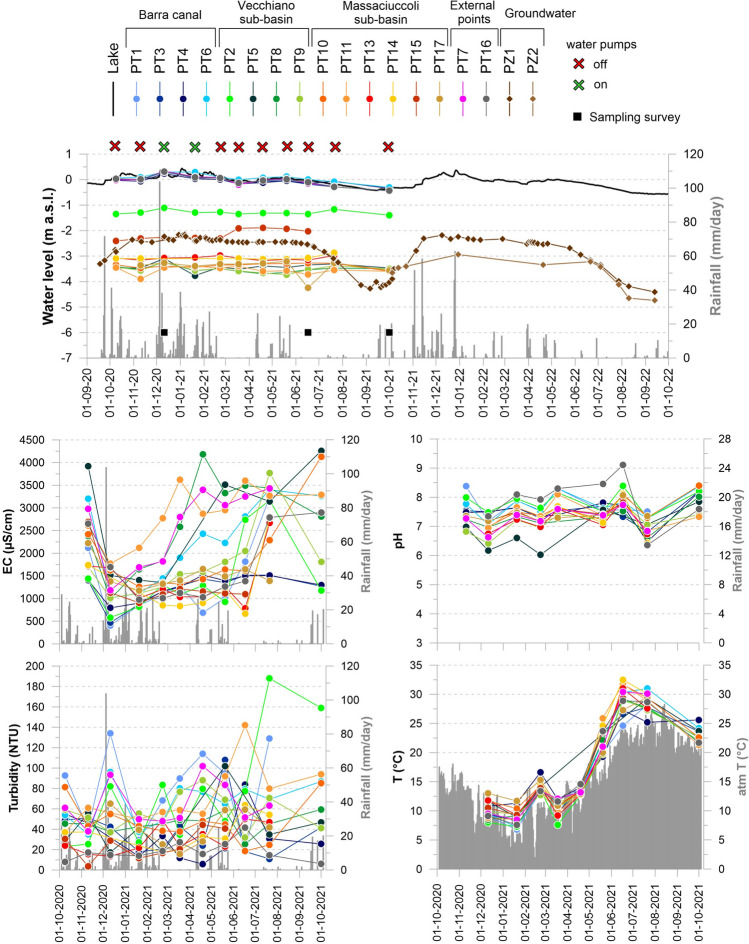
Time series of monitored parameters for surface water (water level, CE, turbidity, pH, T) and groundwater (water level). Data for water level of the Lake Massaciuccoli (m a.s.l.), daily precipitation (mm/day), and the atmospheric temperature (°C) are available at the website of the Regional Hydrologic Service of Tuscany (https://www.sir.toscana.it) and were downloaded from the monitoring stations TOS02004081 (water level of the lake and daily precipitation) and TOS11000001 (atmospheric temperature)

The time series of monitored parameters for surface water and groundwater are reported as summary statistics in Table 1 (raw data are listed in Tables S1 and S2 of supplementary material) and shown in Fig. 3. The water level of the Lake Massaciuccoli (m a.s.l.), the daily precipitation amounts (mm/day), and the atmospheric temperature (°C) as registered by the Regional Hydrologic Service of Tuscany are also reported (Fig. 3).

**Table 1:** Summary statistics of monitored parameters for surface ditches

		Barra canal				Vecchiano sub-basin				Massaciuccoli sub-basin						Ext. points	
		PT1	PT3	PT4	PT6	PT2	PT5	PT8	PT9	PT10	PT11	PT13	PT14	PT15	PT17	PT7	PT16
	min	− 0.27	− 0.44	− 0.35	− 0.30	− 1.40	− 3.77	− 3.69	− 3.73	− 3.59	− 3.90	− 3.18	− 3.15	− 2.43	− 4.25	− 0.27	− 0.44
Water level	max	0.29	0.32	0.23	0.30	− 1.11	− 3.10	− 3.24	− 3.23	− 2.97	− 3.40	− 2.29	− 2.87	− 1.89	− 3.21	0.33	0.31
(m a.s.l.)	mean	− 0.01	− 0.06	− 0.05	0.06	− 1.29	− 3.43	− 3.51	− 3.52	− 3.33	− 3.57	− 3.01	− 3.07	− 2.15	− 3.40	0.00	− 0.03
	sd	0.16	0.19	0.15	0.18	0.09	0.15	0.12	0.13	0.14	0.15	0.24	0.08	0.22	0.28	0.17	0.20
	min	406	468	794	1014	581	1352	992	1013	1259	1771	784	663	906	1176	1183	976
EC	max	3240	1516	1518	3400	3170	4260	4180	3770	4130	3620	2670	2780	2830	2290	3520	2900
(µS/cm)	mean	1298	1204	1253	2266	1370	2729	2644	1734	1920	2923	1386	1298	1458	1574	2645	1843
	sd	870	310	244	900	782	1689	957	712	810	583	639	784	848	571	1097	811
	min	18	11	6	35	23	17	25	32	19	38	14	18	4	16	38	6
Turbidity	max	222	108	84	204	290	102	149	315	169	143	155	169	44	205	155	44
(NTU)	mean	102	39	33	78	92	43	55	76	57	77	39	49	26	55	71	19
	sd	66	25	21	42	78	30	30	74	39	35	39	45	16	52	37	12
	min	7.18	6.81	6.47	7.06	7.29	6.03	6.61	6.42	6.99	6.64	6.74	6.43	7.10	7.18	6.63	6.36
pH	max	8.38	8.20	8.19	8.37	8.39	7.85	8.02	7.79	8.40	8.10	7.90	8.12	7.81	8.07	7.74	9.11
	mean	7.77	7.50	7.52	7.72	7.84	6.90	7.23	7.11	7.54	7.38	7.16	7.27	7.38	7.43	7.22	7.85
	sd	2.37	0.33	0.42	0.45	0.36	3.52	0.41	0.40	0.44	0.46	2.30	3.24	3.12	2.25	2.21	0.74
	min	6.8	8.3	10.8	7.2	7.3	8.9	8.5	9.4	10.4	8.5	9.2	11.2	9.3	10.9	8.5	7.6
T (C°)	max	28.2	27.8	27.3	31.0	28.9	29.3	29.3	29.2	31.7	31.8	31.0	32.5	29.3	29.5	30.4	28.9
	mean	15.7	17.1	17.9	17.6	16.5	18.5	17.1	17.0	17.9	17.7	16.9	20.0	16.2	17.2	17.0	17.1
	sd	8.1	7.2	6.0	8.2	8.1	11.2	7.3	7.0	7.4	8.3	8.8	11.1	8.9	8.1	9.1	7.6

As shown in Fig. 3, the surface water displayed a rather constant water level throughout the monitoring period. As evidenced, Lake Massaciuccoli had the highest water level (ranging between 1 and − 1 m a.s.l.) along with the Barra canal (PT1, PT3, PT4, and PT6) and the external monitoring points (PT7 and PT16), which are in direct connection with the lake (Fig. 2). As regards the other ditches, we can note that the water level decreased from the southeast to the water pumping system. Indeed, PT2 and PT15, which are the southernmost monitoring points, displayed a general higher water level (about − 1 and − 2 m a.s.l., respectively) compared to the other ditches which had the water head ranging between − 3 and − 4 m a.s.l. Throughout the entire monitoring period, even with the water pumping system turned off, the water flow direction of all ditches was indeed generally toward the lake and the water pumps. Even with a minimal monitoring network, we can observe that groundwater displayed a more variable regime compared to surface water, mainly related to precipitation (Fig. 3). In general, both piezometers showed a higher water level than the nearby superficial ditches. PZ1 had a water level higher than PZ2, according to its greater distance from the pumping system (Fig. 2).

While the water level of surface ditches remained rather constant during the monitoring period, the physico-chemical conditions varied considerably (Fig. 3). Specifically, EC and T varied according to precipitation regime and atmospheric temperature, respectively, with lower values in the wettest and coldest season and higher values in the driest and hottest season. pH and especially turbidity appear instead not to be linked to atmospheric conditions. On average, EC showed values higher than 1200 µS/cm, with the highest ones (values > 2000 µS/cm) registered for monitoring points closest to the lake (PT5, PT6, PT7, PT8, and PT11; Table 1). Turbidity was in general higher for the monitoring points located near the water pumps (PT6, PT7, PT8, PT9, PT10, PT11; Fig. 2) and for PT1 and PT2 (Table 1). pH generally varied between 6 and 9 with the lowest values registered for PT5 and the highest for PT16 (Table 1). Temperature ranged from about 7 to 33 °C in accordance with atmospheric temperature (Table 1, Fig. 3).

### Hydrochemistry and water quality

**Fig. 4:**
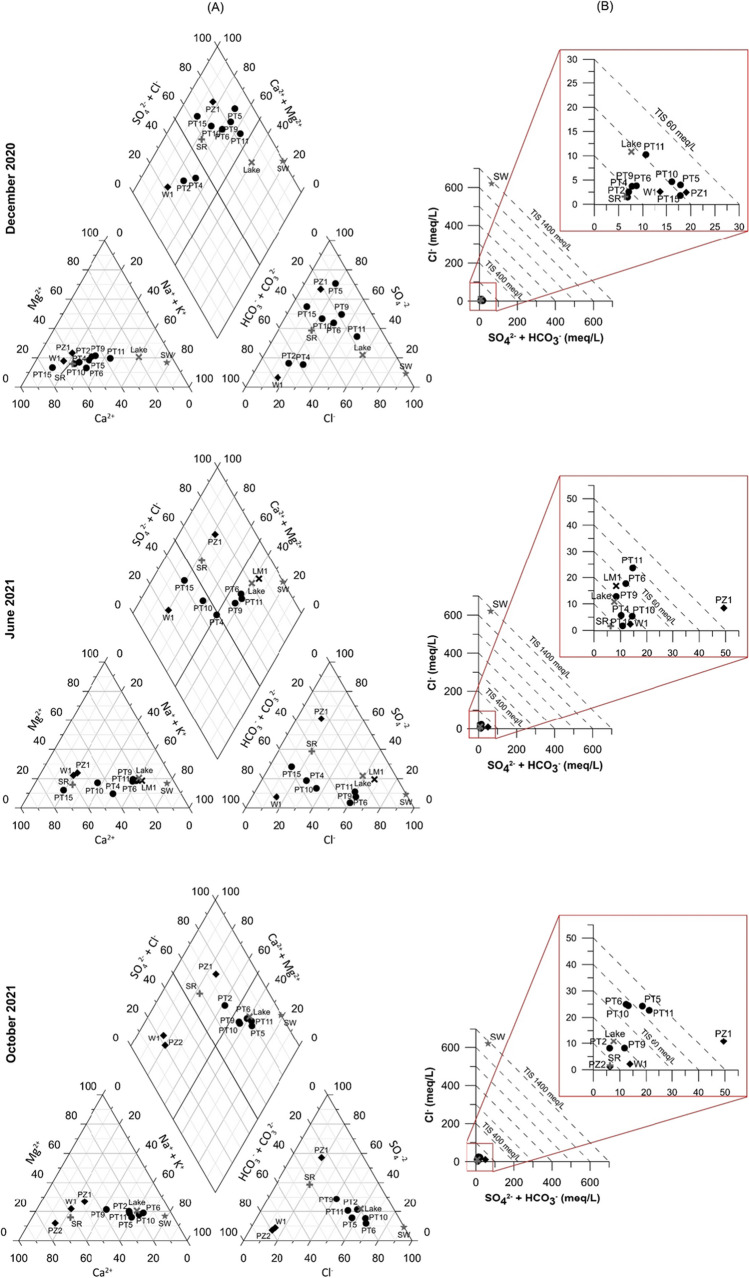
**A** Piper classification diagrams and **B** total ionic salinity (TIS) plots for the three sampling surveys. Samples “Lake”, “SR” and “SW” are literature data for Lake Massaciuccoli (Baneschi 2007), Serchio River (Cortecci et al. 2008) and seawater (Salleolini et al. 2005), respectively

The results of the sampling surveys are listed in Table 2 (field parameters and major ions) and Table 3 (trace elements). Major ions are also plotted in the Piper trilinear diagram to classify water types (Fig. 4A).

**Table 2:** Physico–chemical parameters and concentrations of major ions (mg/L) in water samples collected during the three sampling surveys. For some monitoring points the analyses of June 2021 (PT2 and PT5) and October 2021 (PT4 and PT15) are missing due to the little water in the ditches and therefore the impossibility of sampling

Sample code	Date (dd/mm/yy)	EC (µS/cm)	pH	T (°C)	Ca	Mg	Na	K	Cl	SO_4_	NO_3_	HCO_3_
Barra canal												
PT4	11/12/2020	794	7.5	10.8	101	15	60	15	92	71	27	342
PT4	17/06/2021	1500	7.71	27.3	112	16	146	23	201	103	27	497
PT6	11/12/2020	1014	7.06	9.5	119	27	81	8.5	136	263	25	195
PT6	17/06/2021	2810	7.84	30.3	141	64	371	16	627	158	3.2	534
PT6	01/10/2021	3260	8.37	24.2	126	88	553	24	885	212	< 3	485
Vecchiano sub-basin												
PT2	11/12/2020	581	7.48	8	88	16	42	7.2	53	65	8.7	336
PT2	01/10/2021	1178	8.2	22	77	30	199	19	289	146	< 3	189
PT5	11/12/2020	1512	6.17	11.6	196	52	145	9.2	141	743	35	148
PT5	01/10/2021	4260	7.85	23.7	234	107	583	49	860	320	< 3	723
PT9	11/12/2020	1013	6.42	9.9	102	30	83	9.8	132	273	45	126
PT9	17/06/2021	2050	7.79	29.2	97	49	261	13	454	36	< 3	467
PT9	01/10/2021	1807	7.4	22.7	144	50	178	12	290	275	5.1	369
Massaciuccoli sub-basin												
PT10	11/12/2020	1626	6.99	11.7	237	38	103	6.4	165	466	26	392
PT10	17/06/2021	1615	8.12	31.7	158	36	143	9.1	190	177	< 3	656
PT10	01/10/2021	4130	8.4	22.6	155	85	556	24	867	275	< 3	458
PT11	11/12/2020	1771	6.95	8.8	148	48	195	9.9	363	346	18	209
PT11	17/06/2021	3600	7.82	31.8	167	85	506	18	836	140	3.5	714
PT11	01/10/2021	3290	7.33	21.3	210	105	537	22	804	434	< 3	738
PT15	11/12/2020	1129	7.18	10.5	273	30	49	3.7	64	519	7.5	431
PT15	17/06/2021	1097	7.81	29.3	161	17	49	5.2	60	169	< 3	445
Groundwater												
PZ1	11/12/2020	1556	7.05	12.5	263	43	70	9.5	87	691	34	287
PZ1	17/06/2021	3860	6.7	19.7	579	154	254	14	299	1697	< 3	857
PZ1	01/10/2021	3300	6.96	18.7	559	191	333	21	384	1656	< 3	918
PZ2	01/10/2021	602	7.56	18.6	98	9.8	23	2	37	31	< 3	342
W1	11/12/2020	1131	6.95	13.9	170	41	59	7.4	92	51	< 3	772
W1	17/06/2021	1384	6.92	17	170	40	63	7.6	83	58	< 3	762
W1	01/10/2021	1323	7.04	15.8	176	40	65	8.2	76	55	< 3	775
Lake Massaciuccoli												
LM1	17/06/2021	2800	9.09	/	93	57	351	16	595	236	3.2	210

**Table 3:** Trace elements concentrations (µg/L) of water samples collected during the three sampling surveys. For some monitoring points the analyses of June 2021 (PT2 and PT5) and October 2021 (PT4 and PT15) are missing due to the little water in the ditches and therefore the impossibility of sampling. The values exceeding the Italian threshold of groundwater contamination (D.Lgs. 152/2006, reported in the last row of the table) are highlighted in bold

Sample code	Date (dd/mm/yy)	Li	Be	Mn	Co	Ni	Cu	Zn	Sr	Mo	Ag	Sn	Cd	Sb	Ba	Tl	Pb	Th	U	V	Cr	Fe	As
Barra canal																							
PT4	11/12/2020	21	0.03	**138**	0.52	7.4	4.8	< 40	605	1.1	< 0.2	< 0.3	< 0.04	0.44	38	< 0.1	0.33	0.04	0.8	1.2	0.36	56	1.5
PT4	17/06/2021	46	0.02	**131**	0.59	7.8	6.7	< 40	871	1	< 0.2	< 0.3	< 0.04	0.55	25	< 0.1	0.92	< 0.04	0.08	1.7	0.48	77	2.1
PT6	11/12/2020	14	0.12	**467**	3	16	6.8	< 40	798	< 1	< 0.2	< 0.3	0.06	0.31	42	< 0.1	< 0.3	0.05	0.66	1.2	1	**420**	0.99
PT6	17/06/2021	26	0.06	**457**	0.68	9.9	10	< 40	1199	< 1	< 0.2	< 0.3	< 0.04	< 0.2	53	< 0.1	< 0.3	< 0.04	0.96	3.2	< 0.3	84	3.7
PT6	01/10/2021	13	< 0.02	**275**	0.79	9.4	13	< 40	1151	1.4	< 0.2	< 0.3	< 0.04	0.44	56	< 0.1	< 0.3	< 0.04	1	3.4	< 0.3	117	3.5
Vecchiano sub-basin																							
PT2	11/12/2020	2.5	0.04	**151**	0.54	6.7	4.8	< 40	445	1.3	< 0.2	< 0.3	< 0.04	0.35	48	< 0.1	< 0.3	0.05	1.7	1.2	0.63	34	1.5
PT2	01/10/2021	30	0.03	**419**	0.72	7.1	5.4	< 40	730	1.6	< 0.2	< 0.3	< 0.04	0.75	37	< 0.1	< 0.3	< 0.04	0.75	5.9	< 0.3	65	7
PT5	11/12/2020	8.8	0.15	**274**	1.4	20	12	< 40	1265	< 1	< 0.2	< 0.3	0.09	0.39	48	< 0.1	< 0.3	0.27	0.24	1.3	1.9	**357**	0.81
PT5	01/10/2021	11	< 0.02	**1764**	1.4	16	15	< 40	1841	1.3	< 0.2	< 0.3	< 0.04	0.59	145	< 0.1	0.54	0.06	1.2	5.4	1.5	**328**	8.8
PT9	11/12/2020	23	0.41	**913**	6.3	**31**	6.5	< 40	658	1.1	< 0.2	< 0.3	0.16	0.28	46	< 0.1	0.32	0.13	0.34	1	2.3	**949**	1.1
PT9	17/06/2021	6.7	< 0.02	**466**	0.72	7.6	7.9	< 40	624	< 1	< 0.2	< 0.3	< 0.04	< 0.2	31	< 0.1	< 0.3	< 0.04	0.94	1.8	< 0.3	113	3.5
PT9	01/10/2021	15	0.07	**1119**	2.5	14	4.7	< 40	847	2.1	< 0.2	< 0.3	0.04	< 0.2	38	< 0.1	< 0.3	< 0.04	0.77	1.1	0.59	**239**	1.9
Massaciuccoli sub-basin																							
PT10	11/12/2020	31	0.13	**698**	4.9	**23**	3.7	< 40	1569	< 1	< 0.2	< 0.3	< 0.04	< 0.2	47	< 0.1	< 0.3	< 0.04	1.3	0.67	< 0.3	**608**	0.67
PT10	17/06/2021	58	0.04	**210**	0.55	11	5.2	< 40	1438	< 1	< 0.2	< 0.3	< 0.04	0.2	36	< 0.1	< 0.3	< 0.04	0.69	2.1	< 0.3	52	3.1
PT10	01/10/2021	17	< 0.02	**254**	0.89	11	15	< 40	1374	3.1	< 0.2	< 0.3	< 0.04	0.58	60	< 0.1	0.92	0.09	1.6	2.9	< 0.3	81	2.6
PT11	11/12/2020	17	0.11	**1194**	4.2	20	5.9	< 40	988	< 1	< 0.2	< 0.3	< 0.04	0.47	61	< 0.1	< 0.3	0.08	0.43	2.9	2	**1268**	1.5
PT11	17/06/2021	9	0.06	**805**	1	11	13	< 40	1567	< 1	< 0.2	< 0.3	< 0.04	< 0.2	76	< 0.1	0.56	< 0.04	0.8	5.4	0.73	**247**	3.4
PT11	01/10/2021	15	< 0.02	**1110**	1.3	11	11	< 40	1851	< 1	< 0.2	< 0.3	< 0.04	< 0.2	139	< 0.1	< 0.3	< 0.04	0.14	1.7	0.78	**669**	1.2
PT15	11/12/2020	17	0.03	**546**	1.8	19	3.1	< 40	2684	< 1	< 0.2	< 0.3	0.05	0.26	35	< 0.1	< 0.3	< 0.04	2.7	< 0.6	0.42	51	0.55
PT15	17/06/2021	15	0.06	**1166**	0.71	11	3.4	< 40	1578	1.4	< 0.2	< 0.3	< 0.04	0.43	44	< 0.1	1.9	0.06	1.5	3.6	< 0.3	39	4.9
Groundwater																							
PZ1	11/12/2020	8.1	0.06	**552**	0.97	19	7.2	286	2070	< 1	< 0.2	< 0.3	0.08	< 0.2	38	< 0.1	< 0.3	0.07	1.7	0.63	0.89	63	0.7
PZ1	17/06/2021	16	0.09	**5839**	4.6	**39**	8.5	< 40	5115	< 1	< 0.2	< 0.3	< 0.04	< 0.2	82	< 0.1	4.6	< 0.04	4.3	2.8	2.4	**3146**	2.1
PZ1	01/10/2021	13	< 0.02	**7316**	2.6	**34**	8.7	< 40	4941	< 1	< 0.2	< 0.3	< 0.04	0.31	119	< 0.1	9.3	< 0.04	4.4	3.4	3	**3170**	2.7
PZ2	01/10/2021	3.9	< 0.02	**185**	0.16	4.1	< 2.4	< 40	320	< 1	< 0.2	< 0.3	< 0.04	< 0.2	10	< 0.1	3.9	< 0.04	0.07	0.13	< 0.3	65	0.2
W1	11/12/2020	12	< 0.02	**2404**	0.39	8.7	< 2.4	< 40	1259	< 1	< 0.2	< 0.3	< 0.04	< 0.2	185	< 0.1	< 0.3	< 0.04	< 0.03	< 0.6	< 0.3	**5986**	< 0.2
W1	17/06/2021	10	0.03	**2056**	0.33	8	2.6	< 40	1299	< 1	< 0.2	< 0.3	< 0.04	< 0.2	175	< 0.1	< 0.3	< 0.04	< 0.03	< 0.6	< 0.3	**6219**	< 0.2
W1	01/10/2021	11	0.05	**2162**	0.31	7.2	5.7	< 40	1269	< 1	< 0.2	< 0.3	< 0.04	< 0.2	172	< 0.1	< 0.3	< 0.04	< 0.03	< 0.6	< 0.3	**6158**	< 0.2
Lake Massaciuccoli																							
LM1	17/06/2021	12	< 0.06	12	0.36	7.6	11	< 40	993	< 1	< 0.2	< 0.3	< 0.04	0.41	25	< 0.1	< 0.3	< 0.04	0.76	3	< 0.3	< 32	2.8
D.Lgs. 152/2006		/	4	50	50	20	1000	3000	/	/	10	/	5	5	/	/	10	/	/	/	50	200	10

**Fig. 5:**
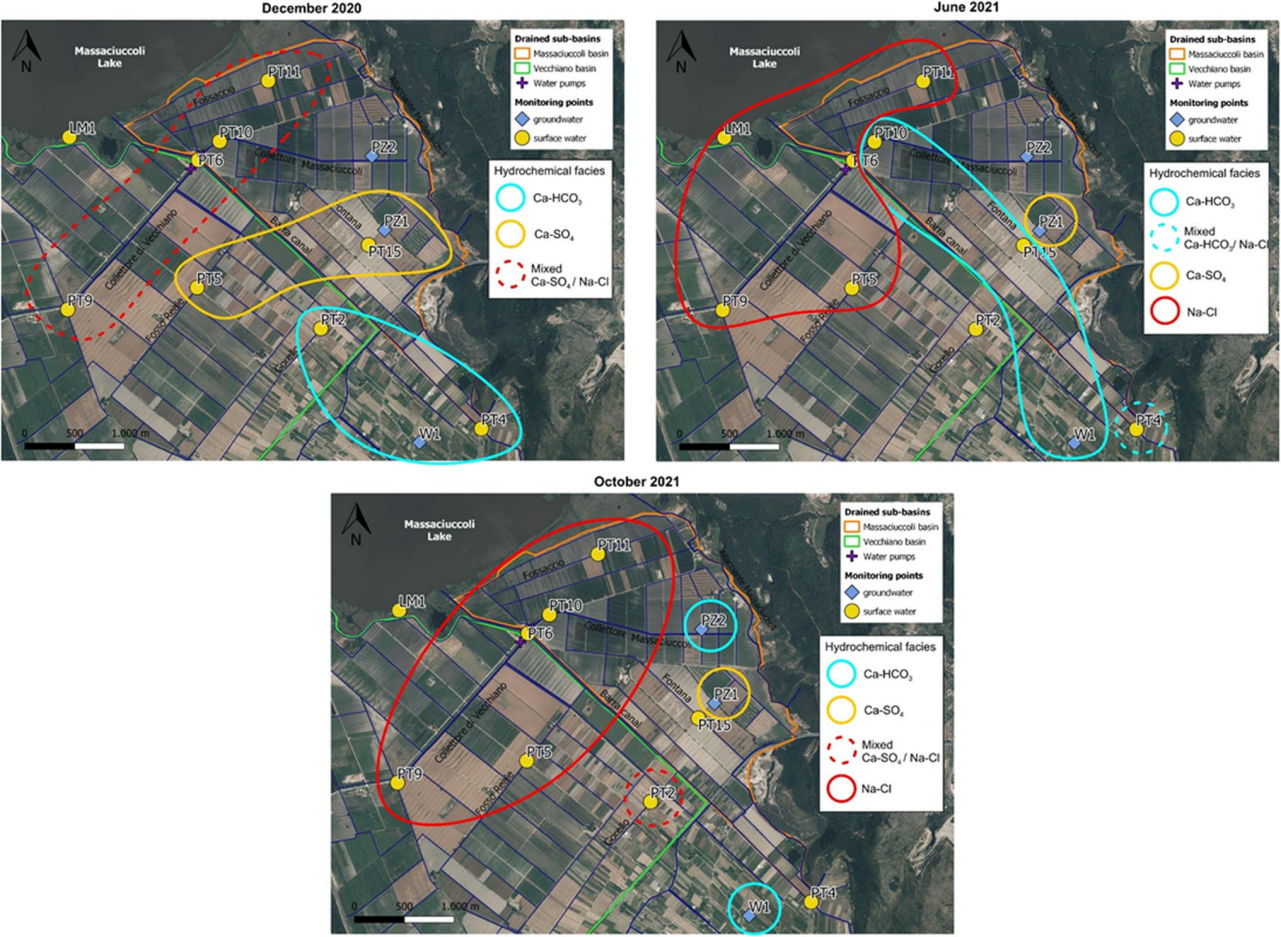
Spatial distribution of hydrochemical facies for the three sampling surveys

As shown in Fig. 4A, three main hydrochemical facies are recognized: (i) Ca-HCO_3_, (ii) Ca-SO_4_, and (iii) Na-Cl. Groundwater showed the same hydrochemical facies in all sampling surveys, namely Ca-HCO_3_ for W1 and PZ2 and Ca-SO_4_ for PZ1. The Lake Massaciuccoli (LM1), sampled in June 2021, had a Na-Cl composition, according to literature data (reported as “Lake” in Fig. 4). In the first survey of December 2020, water samples of surface ditches had mainly a Ca-SO_4_ composition, whereas in the other surveys (June and October 2021) the composition was mainly Na-Cl. Nevertheless, the total ionic salinity (TIS; Fig. 4B) of surface samples was always less than 100 meq/L, and thus very far away from TIS of sea water (reported as “SW” in Fig. 4). Observing the spatial distribution, the hydrochemical facies change from Ca-HCO_3_ to Ca-SO_4_ and Na-Cl moving from the southeastern sector to the lake (Fig. 5). As regards NO_3_, concentrations were variable over time and among samples (Table 2) but were always below 50 mg/L (limit of water quality reported in the Nitrates Directive 91/676/CEE). The highest NO_3_ values were measured in December 2020, both for ditches and groundwater collected at PZ1 (Table 2).

All samples showed trace elements concentrations below the Italian threshold of contamination (D.Lgs. 152/2006), except for Fe and Mn that reach very high values especially in PZ1 and W1 (Table 3). Criticisms were locally observed also for Ni (PT9, PT10, PZ1). Some elements (Zn, Mo, Ag, Sn, Cd, Tl, Pb, Th) resulted below or near the detection limit for all samples in each survey (Table 3). Overall, higher trace elements contents were detected in the surveys sampling of June and October 2021.

**Fig. 6:**
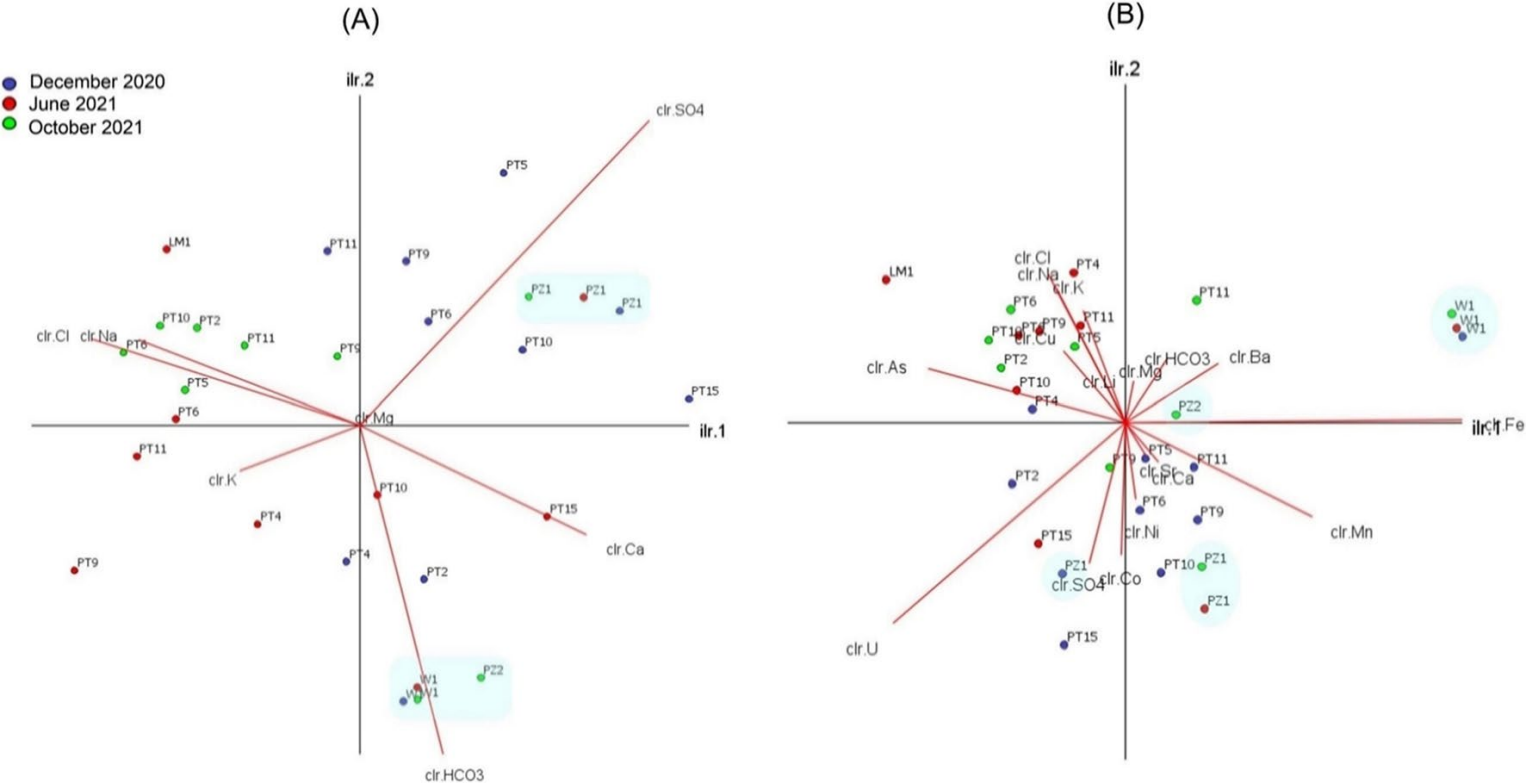
Compositional *clr-*biplots (*α* = 0.5) for **A** major ions (89% of the total variance) and **B** major ions and trace elements (72% of the total variance), with samples categorized for the sampling date. Light blue areas highlight groundwater samples

The *clr*-biplots for the compositional dataset are shown in Fig. 6, whereas the geometric center and variation array are reported in Fig. S2 of the supplementary material. For the sake of clarity, the *clr*-biplot on the left (Fig. 6A) has been realized only with major ions, whereas the *clr*-biplot on the right also includes trace elements (Fig. 6B). The quality of the compositional biplots (Fig. 6) is quite high, since the first two components (ilr.1 and ilr.2) account for about 89% and 72% of the total variance, respectively. Among major ions, the variables with the highest variability (and thus with the longest rays in Fig. 6A) are *clr*-SO_4_ and -HCO_3_, while among trace elements, the most variable are *clr*-Fe and -U (Fig. 6B). According to the low values of variance of the log ratio (Fig. S2) and the shortness of the links in Fig. 6A, among major ions Na and Cl and, to a lesser extent, Ca and HCO_3_ result proportional to each other, and then, they probably carry the same information. Among trace elements, Sr and Ni are proportional to Ca, Cu is proportional to K and Ba is quite proportional to HCO_3_, while Fe, U, and As have high variance of log ratio with all other variables. Samples appear to be distributed in this space according to the sampling date, and thus, samples of December 2020 are mainly distributed toward *clr*-SO_4_, samples of June 2021 are mainly distributed between *clr*-HCO3 and *clr*-Na-Cl, and samples of October 2021 are mainly distributed toward *clr*-Na-Cl (Figs. 6A and 6B). Groundwater samples from different surveys (highlighted in light blue in Figs. 6A and 6B) are instead close to each other.

### Stable isotope composition of water and dissolved sulfates

**Fig. 7:**
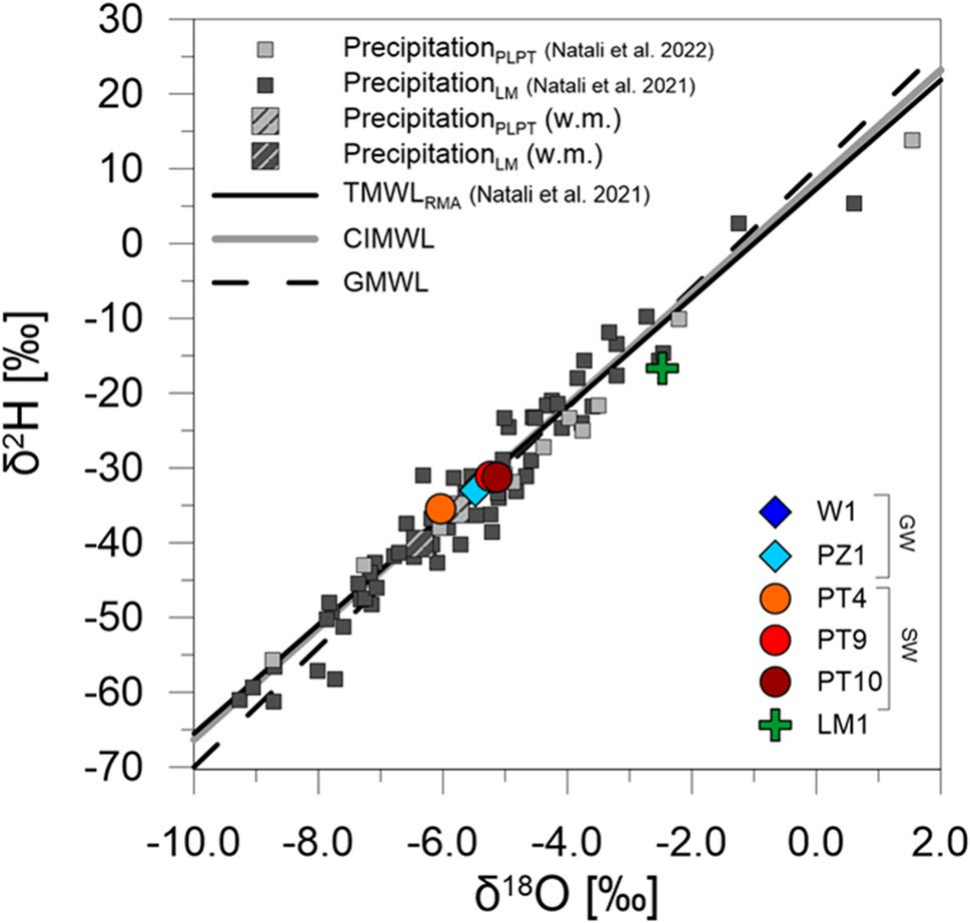
δ^2^H vs. δ^18^O diagram of water samples collected in the Lake Massaciuccoli basin. GW: groundwater; SW: surface water. Isotopic composition of monthly precipitation is also reported for a monitoring site placed on the shore of the lake (LM) in the period 2007–2014 (Natali et al. 2021) and for a rain collector close to Pietrasanta (PLPT, 4.5 m a.s.l.), about 15 km north of the lake, from May 2020 to June 2021 (Natali et al. 2022). Also shown: Global Meteoric Water Line (GMWL, Craig 1961; Rozanski et al. 1993); Central Italy Meteoric Water Line (CIMWL, Giustini et al. 2016); Tuscany Meteoric Water Line (TMWL_RMA_, Natali et al. 2021)

Isotopic data for water samples collected in June 2021 are listed in Table 4. δ^18^O and δ^2^H of water varied, respectively, from a minimum of − 6.04‰ and − 35.5‰ for the southernmost sampling point of surface water (PT4) to a maximum of − 2.47‰ and − 16.6‰ for the Lake Massaciuccoli (LM1). The *d*-excess followed the opposite trend, ranging from a minimum of 3.2‰ for the lake to a maximum of 12.8 for PT4. Groundwater collected in PZ1 and W1 showed the same oxygen and hydrogen isotope composition, because the difference between isotopic values was lower than analytical error. Data in the δ-space (Fig. 7) overlap the Global Meteoric Water Line (Craig 1961; Rozanski et al. 1993) and the regional meteoric line calculated for Tuscany (Natali et al. 2021), except for the lake sample, which is placed below the meteoric lines. Isotope data for precipitation in the study region from previous studies are also reported (Fig. 7) for comparison with surface water and groundwater. Precipitation was monthly collected in the 2007–2014 period on the shore of the Lake Massaciuccoli (Natali et al. 2021), and simultaneously the survey presented in this work (from May 2020 to June 2021) at a site close to Pietrasanta (PLPT, 4.5 m a.s.l.), about 15 km north of the lake (Natali et al. 2022). Surface water and groundwater samples from the Lake Massaciuccoli basin showed isotope values consistent with the amount-weighted mean isotopic composition of precipitation over the survey period (PLPT: δ^18^O_wm_ =  − 5.80‰, δ^2^H_wm_ =  − 35.6‰) and with rainfall collected at the lake a few years earlier (δ^18^O_wm_ =  − 6.37‰, δ^2^H_wm_ =  − 40.2‰). However, groundwater and ditches samples from PT9 and PT10 showed more positive δ^18^O and δ^2^H values. Oxygen and hydrogen isotope ratios in the lake were much more enriched in heavy isotopes compared to stream water and groundwater, and *d*-excess was very low.

**Table 4:** Oxygen and hydrogen isotope ratios of water samples, and sulfur and oxygen isotope ratios of dissolved sulfates in water. Isotope ratios are expressed by the conventional δ‰ notation. The sulfate content (mg/L) in water samples is also reported

Sample code	Date (dd/mm/yy)	δ^18^O-H_2_O (‰)	δ^2^H-H_2_O (‰)	*d*-excess (‰)	SO_4_ (mg/L)	δ^34^S-SO_4_ (‰)	δ^18^O-SO_4_ (‰)
Barra canal							
PT4	17/06/2021	− 6.04	− 35.5	12.8	103	14.4	7.5
Vecchiano sub-basin							
PT9	17/06/2021	− 5.23	− 31.2	10.7	36	7.7	12.8
Massaciuccoli sub-basin							
PT10	17/06/2021	− 5.12	− 31.3	9.7	177	16.3	11.0
Groundwater							
PZ1	17/06/2021	− 5.48	− 33.1	10.8	1697	− 0.6	6.6
W1	17/06/2021	− 5.42	− 32.2	11.1	58	40.8	19.2
Lake Massaciuccoli							
LM1	17/06/2021	− 2.47	− 16.6	3.2	/	/	/

δ^34^S and δ^18^O of dissolved SO_4_ were largely variable among water samples. Groundwater samples exhibited extremely different δ^34^S (and δ^18^O) values, ranging from the minimum of − 0.6‰ (and 6.6‰) for PZ1 to the maximum of 40.8‰ (and 19.2‰) for W1, whereas surface water samples had intermediate values both for δ^34^S and δ^18^O. It is worth noting that the lowest δ^34^S, as measured for groundwater collected at PZ1, was associated with the highest SO_4_ concentration. Conversely, the SO_4_ content was relatively low in W1, which showed the highest δ^34^S.

## Discussion

### Water origin and hydrodynamics

The surface and groundwater samples collected in the Lake Massaciuccoli basin in June 2021 exhibited an oxygen and hydrogen isotope composition within the range of isotope values of precipitation collected in the study area, indicating a meteoric origin of water (Fig. 7). Groundwater samples (PZ1 and W1) showed the same δ^18^O and δ^2^H values, that were consistent, although slightly more positive, with the amount-weighted mean isotope composition of precipitation in the area (PLPT: δ^18^O_wm_ = -5.80‰, δ^2^H_wm_ = -35.6‰, Natali et al. 2022) over the year before the sampling survey (Fig. 7). This indicates a certain degree of homogenization of precipitation, which contribute to groundwater recharge throughout the year. Water mixing and homogenization is also detectable by comparing the isotope values of groundwater with the isotope composition of the most recent rainfall prior to sampling. The last precipitation occurred 24 days before the sampling, and cumulative monthly rainfall amounted to 105 mm in May 2021, with δ^18^O, δ^2^H and *d*-excess values of − 2.20‰, − 10.1‰ and 7.5‰, respectively (Natali et al. 2022). Conversely, groundwater collected at PZ1 and W1 showed lower isotope values, indicating no direct relationship with most recent rainfall and, therefore, the mixing of recharging water over longer periods. Surface water collected at PT9 and PT10 also showed more depleted δ^18^O and δ^2^H values than the most recent rainfall, although their isotope signatures were slightly higher than groundwater. The enrichment in heavy isotopes of surface water of these ditches can be due to evaporation of water drained from the shallow aquifer. The sample collected at PT4 showed the most depleted δ^18^O and δ^2^H value, even lower than groundwater. This suggests a possible mixing in this area with other water sources depleted in ^18^O and ^2^H, such as infiltration from the Serchio River (Cortecci et al. 2008), that flows rather close to this monitoring point (about 1.3 km). Conversely, the Lake Massaciuccoli showed an isotopic signature typical of evaporated lake water, as evidenced by the low *d*-excess and the placement below the LMWL and GMWL (Fig. 7).

**Fig. 8:**
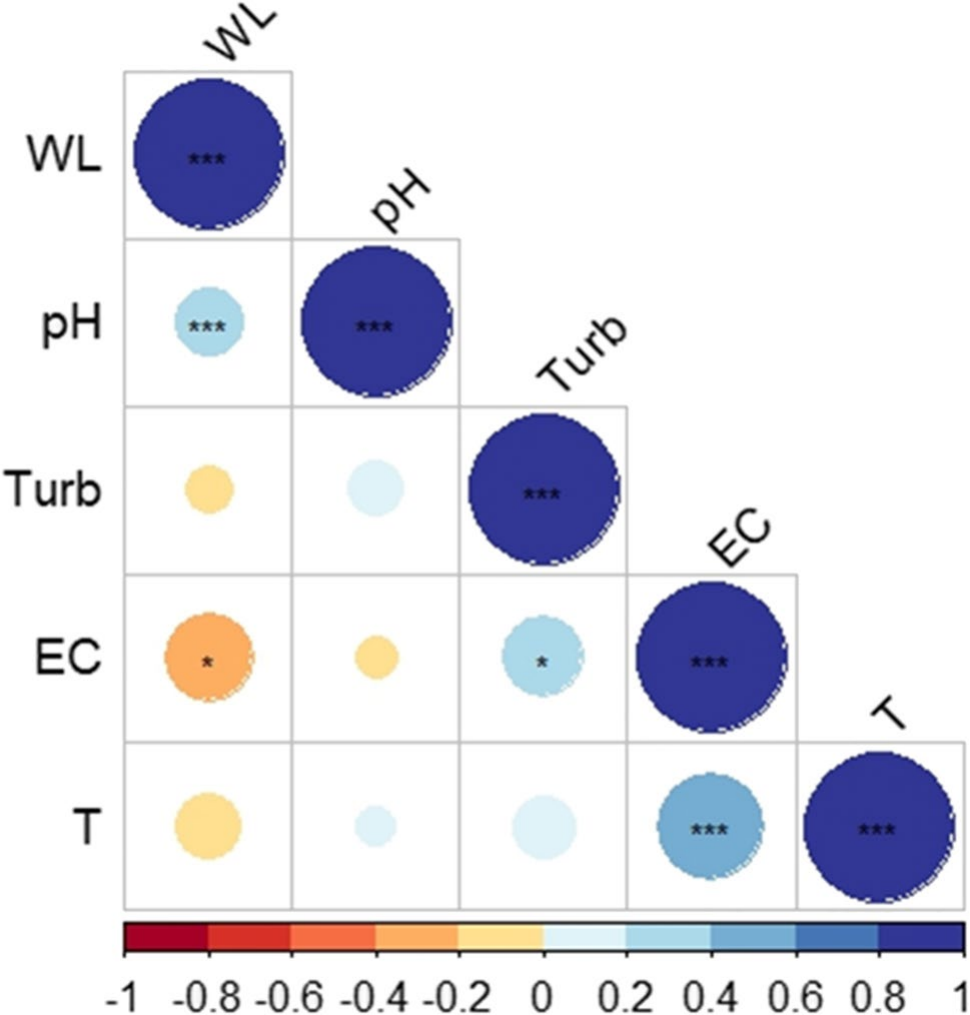
Spearman correlation matrix for field parameters: water level (WL), pH, turbidity (Turb), electrical conductivity (EC) and temperature (T). The number of stars indicates the p-value and thus the level of significance: < 0.05 (*); < 0.01 (**); < 0.001 (***)

The hydrodynamic monitoring indicates that the surface water head and the piezometric level have a lowering gradient from the southeast to the pumping system, in accordance with hydrological and hydrogeological models developed for the area (Rossetto et al. 2010). The higher water level of piezometers compared to the nearby surface ditches indicates that the latter are still able to drain the unconfined aquifer. Moreover, fairly constant surface water level throughout the monitoring period highlights the efficiency of the pumping system in maintaining a stable water level. As shown in Fig. 3, field parameters of surface water varied according to weather conditions, except for turbidity and to a lesser extent pH. Turbidity does not even show any strong relation with other field parameters (Fig. 8) probably because is affected by several factors such as the quantity and shape of suspended particulates, organic matter and microorganisms, the content of dissolved inorganic chemical species and temperature (Kitchener et al. 2017). EC shows the strongest relation with other field parameters, in particular a negative correlation with the water level and a positive correlation with the water temperature (Fig. 8), pointing out its relationship with seasonality and rainfall events: in the driest and warmest season, when the water level is low (due to low precipitation and high evaporation) and the temperature is high, EC tends to increase and vice versa.

### Hydrochemical processes and solutes origin

**Fig. 9:**
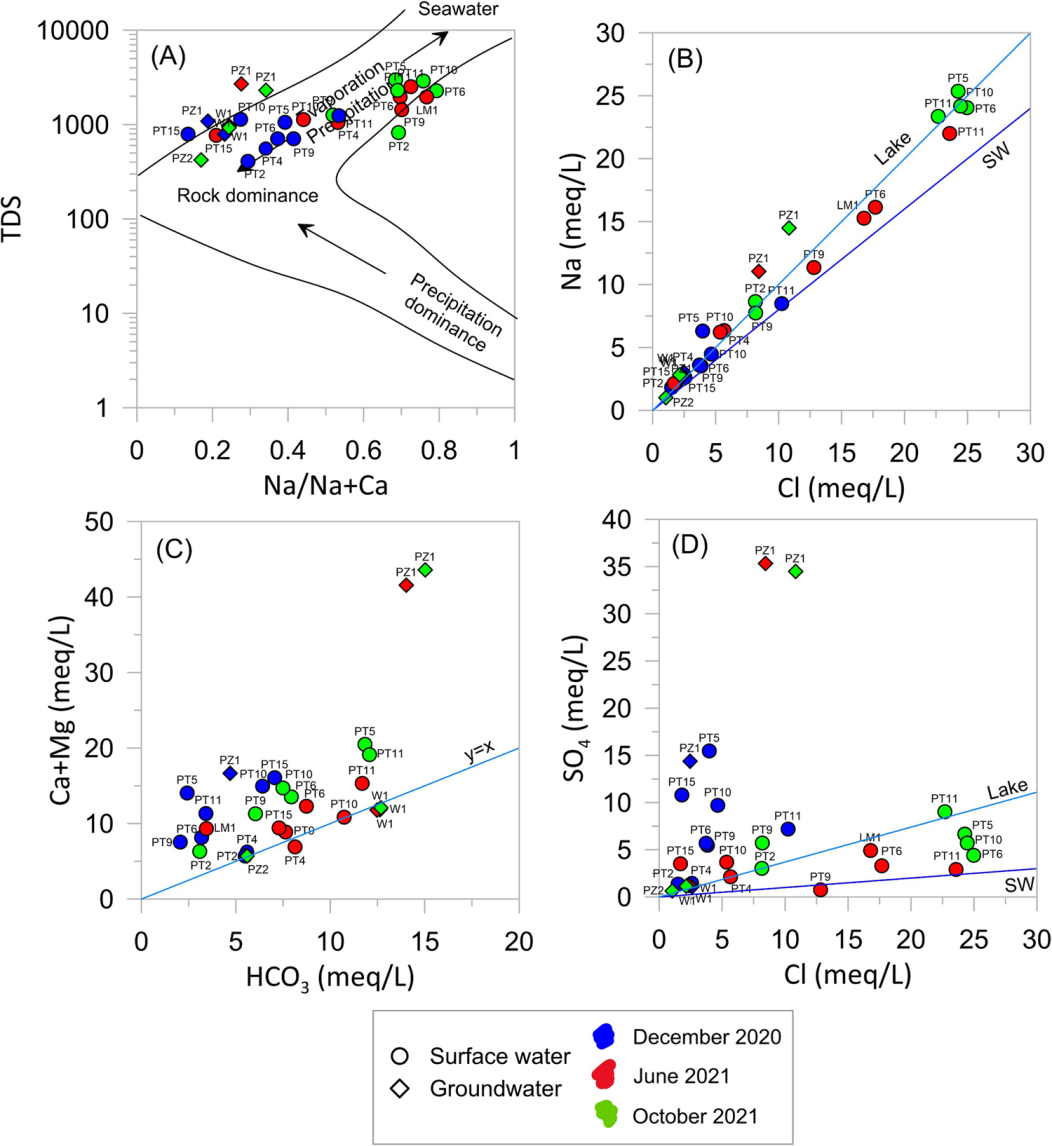
Major ions biplots: **A** Gibbs (1970) diagram showing major processes controlling water chemistry. TDS (mg/L) has been calculated as EC × k where *k* is a constant of proportionality with a value of 0.7 (Taylor et al. 2018); **B** Na vs. Cl; **C** Ca + Mg vs. HCO_3_. The line (*y* = *x*) represents the dissolution of calcite and dolomite; **D** SO_4_ vs. Cl. Reference lines of seawater (SW) and Lake Massaciuccoli (Lake) are from Salleolini et al. (2005) and Baneschi (2007), respectively

The physico-chemical monitoring and chemical analyses indicate that surface water exhibits a high chemical heterogeneity both in time and space, in agreement with previous analysis carried out in the basin (Baneschi et al. 2013). The hydrochemical facies changed from Ca-HCO_3_ to Ca-SO_4_ and Na-Cl moving from the southeastern sector to the lake, tending to become Na-Cl in the hottest and driest season (June and October 2021 surveys) (Fig. 5). On the contrary, groundwater was quite homogeneous in time suggesting that surface water is affected by multiple sources and/or different hydrochemical processes. The Gibbs (1970) diagram (Fig. 9A) suggests that the major processes controlling water chemistry are rock weathering, evaporation/precipitation processes and mixing with seawater. Accordingly, the proportionality between *clr-*Na and *clr-*Cl variables highlighted in the *clr*-biplot (Fig. 6A) and variation array (Fig. S2) suggests that they have the same source, which is likely seawater, as evidenced by classical geochemical biplot (Fig. 9B). Indeed, in the Na vs. Cl biplot (Fig. 9B) samples are distributed between the lines representing the mean composition of seawater (SW) and the Lake Massaciuccoli (Lake), which also has a Na-Cl composition (Fig. 4A) and is connected to the sea through the Burlamacca canal (Fig. 1C). The spatial distribution of Na-Cl water type (Fig. 5) suggests that the mixing between seawater and surface water occurs mostly through the Lake Massaciuccoli. During the hottest and driest season, when precipitation is reduced and the Lake Massaciuccoli is used to supply irrigation, the mixing process is probably enhanced leading surface water of all monitoring points to a Na-Cl composition (Figs. 4A and 5). The mixing process occurs with Ca-HCO_3_ and Ca-SO_4_ water. The proportionality between *clr-*Ca and *clr-*HCO_3_ (Fig. 6A; Fig. S2) suggests that they mainly have the same sources. Groundwater sampled from W1 and PZ2 has also a Ca-HCO_3_ composition and in the diagram of Fig. 9C (Ca + Mg vs. HCO_3_) these samples fall on the line representing the dissolution of calcite and dolomite. This suggests that sources for groundwater, and consequently for the surface water, can be calcite and dolomite present in the soil horizon, carbonate rocks of the eastern reliefs, which are part of the hydrogeological basin (Fig. 1B), and carbonate minerals likely present in the sandy deposits constituting the shallow aquifer. The other samples, and especially PZ1, are distributed above the dissolution line (Fig. 9C), indicating other sources for Ca. An example could be the dissolution of evaporitic gypsum (CaSO_4_) that can be hosted in soil horizons and in the Calcare Cavernoso Fm. (Buchignani et al. 2008; i.e., Boschetti et al. 2011), outcropping on the eastern reliefs (Fig. 1B). Accordingly, gypsum may also be a source of sulfates for the water in the study area. The diagram of Fig. 9D (SO_4_ vs. Cl) suggests indeed other sources for SO_4_ in addition to seawater. Moreover, in the *clr*-biplot (Fig. 6A), sulfates resulted not proportional to any other *clr-*variable, suggesting the existence of multiple sources.

**Fig. 10:**
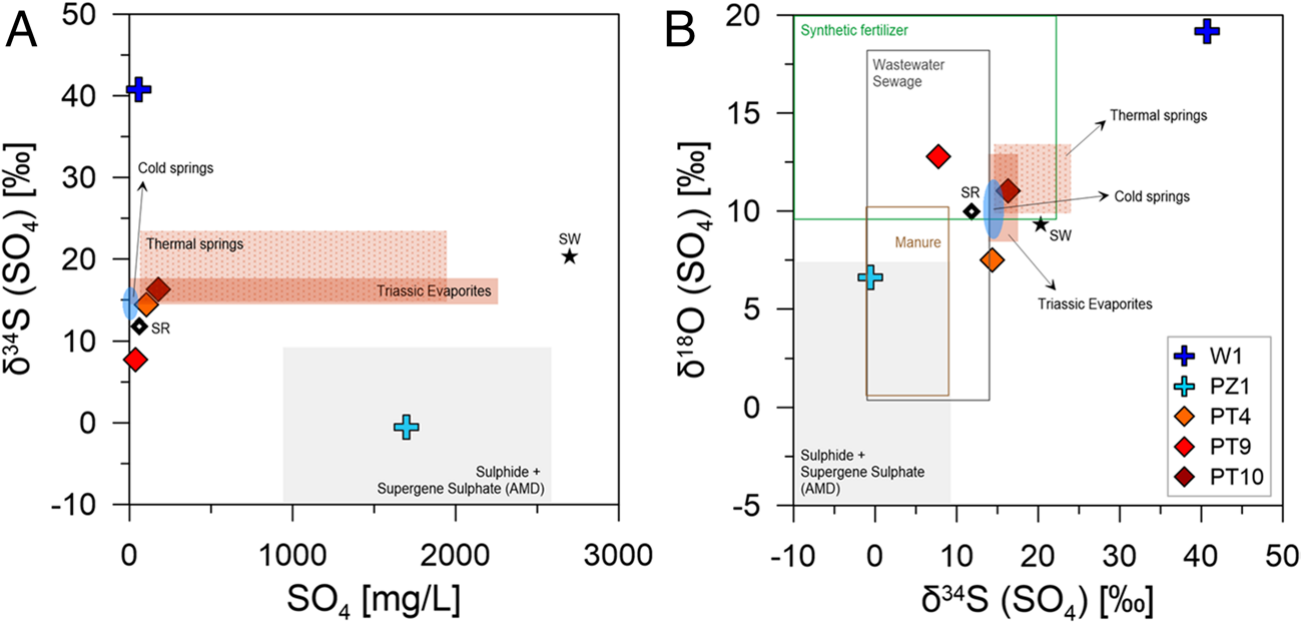
**A** δ^34^S-SO_4_ vs. SO_4_ diagram and **B** δ^34^S-SO_4_ vs. δ^18^O-SO_4_ diagram of water samples collected in the Lake Massaciuccoli basin. SW, seawater; SR, Serchio River; AMD, acid mine drainage. References for isotopic ranges in natural and anthropogenic sulfate sources are given in the text

The stable isotope composition of dissolved SO_4_ has been widely used to recognize different sources and trace the sulfur cycle (Cortecci et al. 2008; Urresti-Estala et al. 2015; Kelepertzis et al. 2023). δ^34^S and δ^18^O of SO_4_ are controlled by (1) the isotopic signature of SO_4_ sources, (2) isotope exchange reactions, and (3) isotope fractionation during biogeochemical processes. Potential sources may have a natural origin (e.g., evaporite dissolution, sulfide oxidation, soil-derived sulfates, and atmospheric deposition) or an anthropogenic origin (e.g., synthetic and organic fertilizers, urban and industrial sewage). Moreover, the sulfate reduction driven by biological activity (bacterial sulfate reduction) (Machel 2001) is the most important kinetic fractionation process in the sulfur cycle, implying a relative enrichment of ^32^S in the reduced product and the consequent depletion of ^32^S in the residual sulfate. The δ^34^S-SO_4_ and δ^18^O-SO_4_ values of water samples are reported in Fig. 10 along with the areas defined by the isotopic ranges of natural and anthropogenic sources. It is worth noting that the isotope signatures for natural sources were compiled by local data, such as Triassic evaporites of Emilia-Romagna and Toscana regions, thermal and cold springs draining evaporitic formations (Boschetti et al. 2005, 2011), Serchio River (Cortecci et al. 2008) and sulfur deposits in northern Apennines and Apuan Alps (Cortecci et al. 1989; Salvioli-Mariani et al. 2024). The δ^34^S and δ^18^O were calculated for the Serchio River as the average of values measured at three sampling sites closer to the Lake Massaciuccoli (S02, S04, S06 in Cortecci et al. 2008). Concerning anthropogenic sources, data were compiled by international references due to lack of local information: acid mine drainage (Migaszewski et al. 2008, 2018; AMD, Gammons et al. 2010; Jakóbczyk-Karpierz and Ślósarczyk 2022); synthetic fertilizers (Vitòria et al. 2004; Zhang et al. 2015; Jakóbczyk-Karpierz and Ślósarczyk 2022); animal manure (Otero et al. 2007; Shin et al. 2014; Jakóbczyk-Karpierz and Ślósarczyk 2022); sewage (Otero et al. 2007; Bottrell et al. 2008; Jurado et al. 2013; Jakóbczyk-Karpierz and Ślósarczyk 2022). Finally, δ^34^S-SO_4_ and δ^18^O-SO_4_ values for seawater are from Tostevin et al. (2016). Groundwater samples showed different δ^34^S (and δ^18^O) values, indicating different local conditions at the sampling sites. PZ1 falls in the fields defined by the isotopic ranges of sulfides (mostly pyrite) and supergene sulfates in AMD (Fig. 10), indicating that for this point, groundwater SO_4_ is mainly controlled by sulfide oxidation. This is consistent with the high content of dissolved SO_4_ in excess of marine sulfate (Fig. 9). According to the characteristics of the study area, pyrite to oxidize could be disseminated within peat deposits. Some studies have shown that coastal peat deposits can be characterized by high enrichment of pyrite due to microbial reduction of seawater sulfate under almost open system conditions (Giblin 1988; Dellwig et al. 2002). Specific studies of peat deposits in this area could validate this hypothesis. Conversely, W1 had very high δ^34^S (and δ^18^O) values, along with low SO_4_, absent NO_3_, and very high Fe and Mn concentrations. These parameters clearly indicate bacterial sulfate reduction at this well, indicating anoxic conditions at deeper levels of the aquifer. Surface water from PT4, PT9, and PT10 showed different δ^34^S and δ^18^O values between PZ1 and W1. PT10 falls in the fields defined by values of Triassic evaporitic sulfate and very close to the Serchio River and cold springs from the same basin. This would suggest an origin of SO_4_ from gypsum dissolution contained in the geological formations of the study area (Fig. 1B). The isotopic shift of PT4 and PT9 suggests the contribution of other sources, such as synthetic fertilizers and/or wastewater. Seawater SO_4_ (Fig. 10) probably constitutes a small part of the total sulfate dissolved in ditches, while SO_4_ contribution from precipitation is negligible due to very low concentrations in rainfall (1.8 ± 1.5 mg/L, Tab. S3) collected at about 15 km north of the lake (PLPT) in the period May-December 2020 (Natali et al. 2022), which would contribute to a maximum of ~ 5% of the total dissolved SO_4_ in surface and groundwater.

As regards NO_3_, concentrations below the limit of water quality reported in the Nitrates Directive 91/676/CEE (50 mg/L) reflect the less intense agricultural practices implemented in recent years (Baneschi et al. 2013). However, remarkably higher NO_3_ values detected in the field survey of December 2021 than in other surveys (Table 2) can be attributable to increased leaching of soil by autumn-early winter rains, as also reported in previous investigations (Pistocchi et al. 2012).

Trace elements show in general a low concentration, except for Fe and Mn that reach very high values especially in groundwater (Table 3). These elements seem not strictly related to other trace elements or major ions (Fig. 6B), suggesting they are affected by different processes and/or sources. Usually, high contents in Fe and Mn in shallow aquifers are found in areas with Fe–Mn mineral rich-strata and soil with abundant organic matter affected by water oscillation and changing in redox conditions (Palmucci et al. 2016; Hamer et al. 2020; Zhai et al. 2021). Fe and Mn are indeed redox-sensitive elements, and the vertical movement of water in the aquifer can promote the degradation of organic matter in oxygenated environments and the subsequent release of Fe and Mn in water under reducing conditions (Palmucci et al. 2016). Inputs from agricultural activities also increase Fe and Mn concentration in water (Zhai et al. 2021). Therefore, in the study area, it is plausible that the degradation of peat also favored by reclamation operations, and the agricultural activities are the main causes of the high concentration of Fe and Mn in surface water and groundwater. The lower contents in surface water can be due to oxygenated conditions which favor the precipitation of Fe/Mn oxides. However, further studies are needed to clearly disentangle the factors and mechanisms responsible for Fe and Mn contamination. Also Ni slightly exceeds the Italian threshold of contamination (D.Lgs. 152/2006) in some samples (Table 3). In this case, this element appears to be proportional to Ca and Sr (Fig. 6 and Fig. S2) suggesting that it may have the same sources or has been affected by the same processes.

## Conclusion

In this study, we provided new and updated data about the hydrological and chemical-physical conditions of surface water and groundwater of one of the largest and most important residual coastal wetlands in Tuscany, highly affected by human activities.

Our results indicated that the hydrodynamic conditions are almost unchanged compared to the latest studies, with water flowing from the southeast to the Lake Massaciuccoli, also depending on the management of reclamation activity. Water stable isotopes indicated that both surface water and groundwater have a meteoric origin, pointing out the presence of geochemical processes affecting water chemistry. Geochemical characteristics of groundwater resulted variable in space but not in time, indicating local geological variability and processes affecting groundwater composition such as degradation of peat and pyrite and bacteria-mediated redox processes. On the other hand, surface water showed geochemical variability in both time and space, indicating that, compared to groundwater, additional sources and processes affect surface water, such as sea water mixing through the Lake Massaciuccoli and evapotranspiration/precipitation processes. Overall, it is not easy to disentangle the origin of sulfur in this complex wetland with multiple sources and variable redox conditions, but geochemistry coupled with isotopes can provide useful insights especially when data is compared with site-specific sources. The impact of fertilizer use on the water quality appears to be limited as regards nitrates, indicating that less intense agricultural practices implemented in recent years have been successful. As regards sulfates, Fe, and Mn, we cannot fully elucidate the mechanisms underlying human influence, but the oscillation of water level and degradation of peat enhanced by reclamation and agriculture activities likely played an important role in controlling the fate of these elements. This points out that the participation of farmers and local stakeholders in the management and planning actions of the area are fundamental in order to adapt socio-economic needs with the restoration and preservation of the area. Policy-making authorities should take actions as soon as possible to mitigate risks, also throughout closer co-operation with farmers to reduce inputs of fertilizers and chemicals into the lake and the surrounding area. Also, additional policy measures should be enforced to reduce the mechanical soil tillage and limit erosion and runoff, such as the NBSs implemented within the Phusicos Project. However, the role of human activities in high sulfate, iron, and manganese contents should be deepened.

Overall, this study shows how the integration of hydrochemical data, stable isotope hydrology, and multivariate statistics can assist in identifying hydrochemical patterns, processes, and interactions between groundwater and surface water and the origin of solutes and their evolution. However, this type of studies based on traditional monitoring can be improved thanks to the development of the Internet of Things, which allows to implement high-frequency and low-cost monitoring and thus increase the amount of data. Moreover, to further deepen the knowledge in this area and the interactions between soil and groundwater, future research should be aimed at evaluating the role of peat degradation as sources of nutrients and other elements in groundwater. Moreover, detailed studies of peat characteristics and peat degradation can also give new insights on subsidence processes and carbon dioxide release in the atmosphere.

### Supplementary Information

Below is the link to the electronic supplementary material.

Supplementary file1 (XLSX 25.4 KB)

Supplementary file2 (PDF 496 KB)

## Data Availability

All data are already available as supplementary material.
