# Supplementary material for: Hydrodynamics and water quality of a highly anthropized wetland: the case study of the Massaciuccoli basin (Tuscany, Italy)
Source: Environ Sci Pollut Res Int. 2024 Jun 18;31(30):43117–37. doi: 10.1007/s11356-024-33899-2 (PMC11222230; doi:10.1007/s11356-024-33899-2)
Supplement: Supplementary file 2 — Supplementary file2 (PDF 496 KB) [file 11356_2024_33899_MOESM2_ESM.pdf]

Supplementary Figures

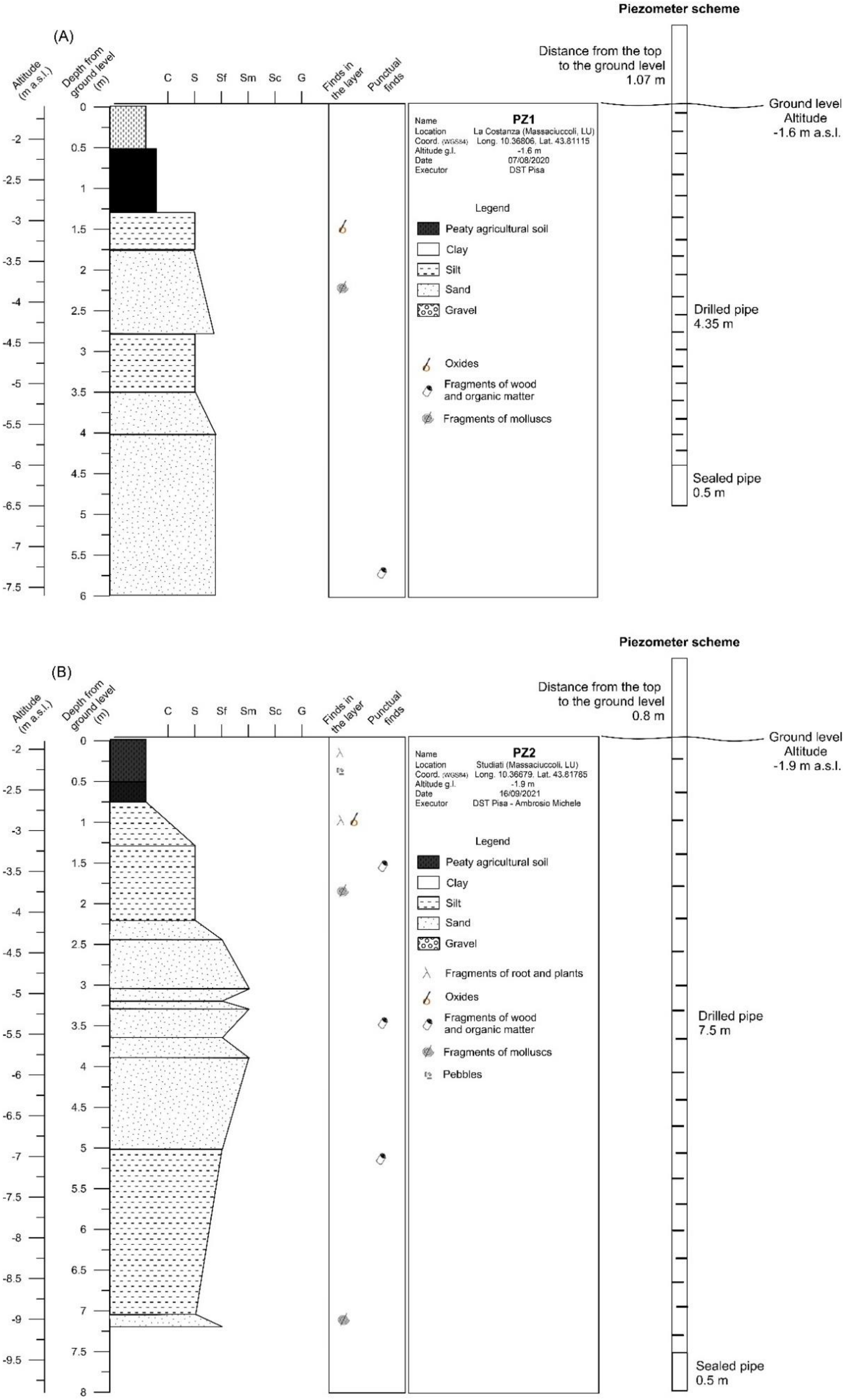

Figure S1. Stratigraphy and scheme of the piezometers installed for the drilling (A) PZ1 and (B) PZ2. In the stratigraphy sections the letters C, S, Sf, Sm, Sc and G mean Clay, Silt, Sand fine, Sand medium, Sand coarse and Gravel, respectively.

Statistics

	Center
Ca	0.1319
Mg	0.0359
Na	0.1208
K	0.0094
Cl	0.1748
SO4	0.1665
HCO3	0.3431
Li	0.0001
Mn	0.0048
Co	0.0000
Ni	0.0001
Cu	0.0001
Sr	0.0099
Ba	0.0004
U	0.0000
Fe	0.0021
As	0.0000

Variation array:

	Variance ln(Xi/Xj)																	
Xi\Xj	Ca	Mg	Na	K	Cl	SO4	HCO3	Li	Mn	Co	Ni	Cu	Sr	Ba	U	Fe	As	clr variances
Ca		0.2975	0.9067	0.5917	1.0759	0.5778	0.2566	0.6115	1.0310	0.6856	0.1514	0.5209	0.0589	0.3636	2.0459	2.3237	1.6212	0.1473
Mg	-1.3007		0.2913	0.3408	0.3976	0.6757	0.4730	0.8603	1.3179	0.8332	0.3934	0.3061	0.2499	0.3958	2.0844	2.4056	1.3051	0.1184
Na	-0.0875	1.2132		0.2468	0.0233	1.1723	0.9059	0.9747	2.3891	1.2611	0.9251	0.2743	0.7991	0.9486	2.1710	3.7148	0.7742	0.4213
K	-2.6380	-1.3373	-2.5505		0.3156	1.1232	0.5630	0.5950	2.0005	1.0632	0.6130	0.1581	0.5751	0.5498	2.1959	3.2792	0.7670	0.2566
Cl	0.2815	1.5822	0.3690	2.9195		1.3704	1.0302	1.0471	2.5881	1.3401	1.0610	0.3670	0.9780	1.0903	2.2746	3.8957	0.8078	0.5322
SO4	0.2333	1.5340	0.3208	2.8713	-0.0482		1.4434	1.1450	1.9009	0.5021	0.3871	0.8605	0.4985	1.3069	1.4663	3.4541	1.6756	0.5261
HCO3	0.9559	2.2566	1.0434	3.5939	0.6745	0.7227		0.7111	1.2436	1.3889	0.6368	0.6841	0.3194	0.3296	2.7173	2.5393	1.6891	0.3715
Li	-7.0384	-5.7377	-6.9508	-4.4003	-7.3198	-7.2716	-7.9943		2.0648	0.9176	0.5347	0.7100	0.5968	0.8819	2.5347	3.3573	1.3008	0.4840
Mn	-3.3226	-2.0219	-3.2351	-0.6846	-3.6040	-3.5559	-4.2785	3.7158		1.4969	1.2103	2.1874	1.1122	0.9024	4.0469	1.1209	3.4789	1.1457
Co	-9.6596	-8.3589	-9.5720	-7.0216	-9.9410	-9.8928	-10.6155	-2.6212	-6.3370		0.2493	0.8912	0.7647	1.1419	1.7145	2.8262	1.6278	0.4758
Ni	-7.1844	-5.8837	-7.0969	-4.5464	-7.4659	-7.4177	-8.1404	-0.1461	-3.8619	2.4751		0.4734	0.2200	0.5570	1.7444	2.4840	1.4200	0.1438
Cu	-7.8552	-6.5545	-7.7676	-5.2171	-8.1366	-8.0884	-8.8111	-0.8168	-4.5326	1.8044	-0.6707		0.5227	0.6858	1.8329	3.5468	0.7799	0.2462
Sr	-2.5860	-1.2853	-2.4984	0.0521	-2.8674	-2.8192	-3.5419	4.4524	0.7366	7.0736	4.5985	5.2692		0.3878	1.9613	2.4855	1.5175	0.1431
Ba	-5.6847	-4.3840	-5.5972	-3.0467	-5.9662	-5.9180	-6.6406	1.3536	-2.3621	3.9749	1.4997	2.1704	-3.0988		3.2038	1.6280	2.0442	0.3413
U	-10.3311	-9.0304	-10.2436	-7.6931	-10.6125	-10.5644	-11.2870	-3.2927	-7.0085	-0.6715	-3.1466	-2.4759	-7.7451	-4.6464		7.3872	1.1645	1.7606
Fe	-4.1408	-2.8401	-4.0533	-1.5028	-4.4223	-4.3741	-5.0967	2.8976	-0.8182	5.5188	3.0436	3.7143	-1.5548	1.5439	6.1903		6.1677	2.4706
As	-9.3551	-8.0544	-9.2675	-6.7171	-9.6365	-9.5883	-10.3110	-2.3167	-6.0325	0.3045	-2.1706	-1.4999	-6.7691	-3.6704	0.9760	-5.2143		1.0309
	Mean ln(Xi/Xj)																	10.6155 Total Variance

Figure S2. Compositional statistics summary: geometric center (on the left) and variation array (on the right). In the variation array variances below the 5th percentile are highlighted in dark blue, from 5th to 25th percentile in light blue, from 75th to 95th percentile in light red, and above the 95th percentile in dark red.
